# Magnetic nanoparticle contrast agents for MRI: structure-property relationships, *in vivo* applications, and future theranostic directions

**DOI:** 10.1088/1361-6528/ae4e33

**Published:** 2026-03-16

**Authors:** Bahareh Rezaei, Shahriar Mostufa, Karla Mercedes Paz González, Ebrahim Azizi, Changzhi Li, Jenifer Gómez-Pastora, Rui He, Kai Wu

**Affiliations:** 1 Department of Electrical and Computer Engineering, Texas Tech University, Lubbock, TX 79409, United States of America; 2 Department of Chemical Engineering, Texas Tech University, Lubbock, TX 79409, United States of America

**Keywords:** magnetic resonance imaging, medical imaging, contrast agents, magnetic nanoparticles, gadolinium

## Abstract

Magnetic resonance imaging (MRI) is a non-invasive and non-ionizing imaging modality that provides high-resolution images of internal organs such as the breast, brain, and cardiovascular system, enabling three-dimensional visualization of soft tissues. While MRI offers excellent soft tissue contrast, its sensitivity can be further enhanced using contrast agents, and many clinical applications rely on exogenous agents to improve detection and diagnostic accuracy. Two primary classes are used clinically: paramagnetic substances, exemplified by gadolinium (Gd), which predominantly shorten longitudinal (*T*_1_) relaxation, and superparamagnetic iron oxide nanoparticles (SPIONs), which exert strong effects on transverse (*T*_2_) relaxation. The performance and safety of these agents are strongly influenced by their pharmacokinetics and biodistribution, including rapid recognition and clearance by the reticuloendothelial system, which can both enable liver–spleen imaging and limit target-specific contrast in other organs. In this review, we first summarize the fundamental principles of MRI contrast generation, with an emphasis on relaxation mechanisms relevant to magnetic nanoparticles (MNPs). We then discuss the use of MNPs as contrast agents in representative biomedical applications, focusing on cardiac, breast, and brain MRI and illustrating how organ-specific physiology constrains nanoparticle design and performance. Finally, we examine biocompatibility and safety considerations for both Gd-based agents and SPIONs, highlighting current regulatory concerns, open questions regarding long-term toxicity, and key challenges that must be addressed to translate next-generation nanoparticle-based MRI contrast agents into routine clinical practice.

**Abbreviations**
BBB: blood-brain barrier
BCS: breast-conserving surgery
BSA: bovine serum albumin
BM: brain metastases
CE-MRI: contrast-enhanced MRI
CMR: cardiovascular MRI
CT: computed tomography
DCE: dynamic contrast-enhanced
DMCAs: dual-mode contrast agents
FID: free induction decay
FLAIR: fluid-attenuated inversion recovery
Gd: gadolinium
GE: gradient echo
Gd_2_O_3_: gadolinium oxide
Gd-DTPA: gadolinium-diethylenetriamine-pentaacetic acid
MRA: magnetic resonance angiography
MRI: magnetic resonance imaging
MR: magnetic resonance signal
MI: myocardial infarction
NAC: neoadjuvant chemotherapy
NSF: nephrogenic systemic fibrosis
NMR: nuclear magnetic resonance
pCR: pathological complete response
PET: positron emission tomography
PSIR: phase-sensitive inversion recovery
PLA: poly (D, L-lactide)
PLGA: poly (D, L-lactide-co-glycolide)
PGA: poly (glycolide)
PEG: polyethylene glycol
RT: radiation therapy
RES: reticuloendothelial system
RF: radiofrequency
ROIs: regions of interest
*r*_1_
: longitudinal relaxivity
*r*_2_
: transversal relaxivity
SE: spin echo
SPECT: single-photon emission computed tomography
*T*_1_
: longitudinal
*T*_2_
: transverse
UI: user interface
ZDS: zwitterionic dopamine sulfonate

## 1. Introduction

The visualization of human tissues and organs to assess normal and abnormal anatomy and physiology is known as medical imaging. A variety of medical imaging methods, including CT [1, 2], PET [3], MRI [4–7], digital mammography [8], SPECT [9], and x-rays [10], are employed for this purpose. Advanced medical imaging techniques can be used to diagnose a wide range of medical ailments, including congenital heart disease, neurological disorders, cardiac diseases, various types of cancers [11], gastrointestinal illnesses, and complex bone fractures [12, 13]. Among all these imaging methods, MRI [14–16] has dramatically advanced since it was first used in clinical settings; its advancements have been linked to a growing variety of clinical, technical, and scientific applications [17, 18].

MRI is a non-invasive medical imaging technique that uses strong magnetic fields and RF pulses to generate detailed, multiplanar, and realistic three-dimensional images based on the magnetic relaxation behavior of water protons in the soft tissues of almost every internal structure in the human body [7, 19–22]. MRI provides high-resolution images of soft tissues, which are often in the order of millimeters, aiding in diagnosing and monitoring various medical conditions. It is distinguished among imaging approaches because it does not use ionizing radiation and is associated with fewer harmful side effects [21, 23–25]. Additionally, MRI can capture dynamic physiological changes while providing excellent soft tissue contrast and high spatial resolution in tomographic three-dimensional datasets [26–28]. MRI has evolved from a method once acknowledged for its enormous potential to one of the most valued and important diagnostic imaging modalities, and its use in clinical practice is increasing due to lower costs and improved accessibility [17, 28, 29]. NMR, which is frequently employed by chemists to determine a substance’s chemical structure, is the main source of MRI [30]. In 1973, Paul C. Lauterbur released images showing the NMR response of hydrogen nuclei in a pair of water-filled glass capillaries, which broadly introduced MRI to the scientific world [31]. In the 1970s, Peter Mansfield modified this method and refined the mathematical analysis of MR signals by introducing echo-planar imaging, which enabled faster image acquisition and improved image quality [32]. Together, these developments marked a major turning point in the evolution of MRI, transforming it from a laboratory method into a powerful clinical imaging tool capable of providing detailed anatomical and functional information.

Several recent reviews have studied magnetic nanoparticles (MNPs) for imaging, but with scopes that differ from the present work. For example, Farinha *et al* [33] provided a broad diagnostic overview of MNPs across imaging and molecular diagnosis, with extensive emphasis on magnetic separation-based assays and MRI considered alongside these other applications rather than as the main organizing theme. Mishra and Yadav’s work [34] focused primarily on synthesis routes, surface chemistry, and general biomedical uses of MNPs (drug delivery, hyperthermia, and imaging), with MRI being a subsection rather than the organizing framework. Mao *et al* [16] reviewed functional nanoparticles for MRI, emphasizing targeting and multifunctionality, but largely from a pre-2016 perspective and without an organ-specific discussion of design constraints. Lapusan *et al* [15] focused on superparamagnetic iron oxide nanoparticles (SPIONs) in MRI from a translational and clinical trial viewpoint, mapping preclinical and clinical studies, but they did not cover MRI physics or contrast mechanisms in detail, and only briefly touched on non-cardiac organ applications. In contrast, our review is explicitly MRI-centric: we begin with basic MRI and relaxation principles, then relate core size, composition, and surface coating of MNPs to *T*_1_/*T*_2_ contrast behavior, and finally organize the *in vivo* discussion around representative organ systems (cardiac, breast, and brain), integrating biocompatibility, regulatory experience with both Gd-based agents and SPIONs, and a forward-looking perspective on where MNPs are most likely to add clinical value.

In this review, we first introduce the fundamentals of MRI and MRI contrast agents in section 2, with an emphasis on the relaxation mechanisms that are particularly relevant for MNPs. Several comprehensive reviews have previously discussed MNPs for MRI and related diagnostic applications; however, most either focus predominantly on clinical trial pipelines or treat MRI as one of several imaging modalities rather than analyzing organ-specific design considerations. Here, we take a complementary, application-driven perspective. In section 3, we discuss the use of MRI in molecular and cellular imaging, focusing on cardiac, breast, and brain disorders as representative examples in which nanoparticle-based contrast agents can improve the detection and characterization of disease processes. We then comment on the biocompatibility and safety concerns associated with the use of MRI contrast agents, including MNPs, in section 4. Finally, in section 5, we provide our perspective on the key challenges and future advancements needed to translate MNP-based MRI contrast agents into routine clinical practice.

## 2. Fundamentals of MRI and MRI contrast agents

### 2.1. MRI scanner

**Figure 1.**
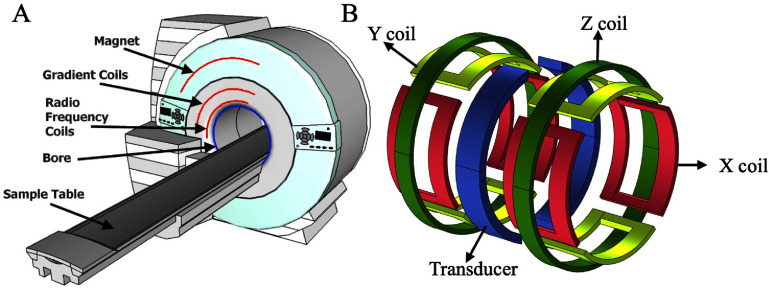
(A) Conceptual schematic provides a comprehensive perspective of an MRI system. (B) A diagram illustrating the setup of the MRI magnetic gradient coils: saddle coil pairs make up the *x*- and *y*-axes, whereas Maxwell coil pairs make up the *z*-axis. (A) Reproduced from [37]. CC BY 3.0. (B) Is an original figure prepared by the authors.

The schematic of an MRI scanner is shown in figure 1(A); it contains four key components that together define the magnetic and RF environment experienced by hydrogen nuclei during imaging. The first is a magnet that produces a strong static magnetic field (denoted by *B*_0_) necessary for proton nuclear polarization [35, 36]. These magnets typically generate fields ranging from 1.5 to 3.0 T and are immersed in liquid helium to become superconductive, thereby minimizing electrical resistance and energy losses [35]. The strength and homogeneity of *B*_0_ are critical parameters because they directly influence spin polarization, resonance frequency, and the relaxation behavior of both tissues and exogenous contrast agents. As shown in figure 1(B), the static field *B*_0_ is generated by a pair of Helmholtz coils aligned along the symmetry *z*-axis of the MRI bore. Additionally, three orthogonally arranged gradient coils, two pairs of saddle coils, and one pair of Maxwell coils create linear magnetic field gradients along the *X, Y*, and *Z* axes, allowing spatial encoding of the MR signal. The second key component is a RF system, which generates the alternating magnetic field (*B*_1_) at the resonance frequency and detects the resulting magnetic resonance (MR) signal emitted by tissue. Short RF pulses tilt the net magnetization away from the *B*_0_ direction, and the precessing transverse magnetization is then detected by RF receive coils tuned to the Larmor frequency. The geometry and positioning of these coils determine the local signal-to-noise ratio and, consequently, the sensitivity to contrast changes produced by administered agents. The third component is the gradient coil system, responsible for producing spatially varying magnetic fields that overlay *B*_0_. These gradient coils are positioned orthogonally in the *X, Y*, and *Z* directions and are concentrically placed within the bore alongside the whole-body RF coils in clinical MRI systems. By superimposing small, linear variations in the magnetic field on top of *B*_0_, gradients encode spatial information into the phase and frequency of the MR signal, enabling slice selection and image formation without directly generating contrast themselves [30].

The final component is made up of computers that offer the UI, convert the patient signals that have been collected from analog to digital, and produce visuals that can be seen and understood on the console. After the acquisition, quantitative relaxation maps produced by the computer systems can be presented on the scanner interface. The advantages of quantification include the ability to conduct more accurate and exact measurements and diagnoses, as well as perform basic research on the biological changes in specific diseases and their response to prospective treatments [36]. For contrast-agent development, including MNPs, these quantitative maps also provide a practical framework for comparing relaxivity and *in vivo* performance across different pulse sequences and field strengths.

### 2.2. Working principles of MRI

**Figure 2.**
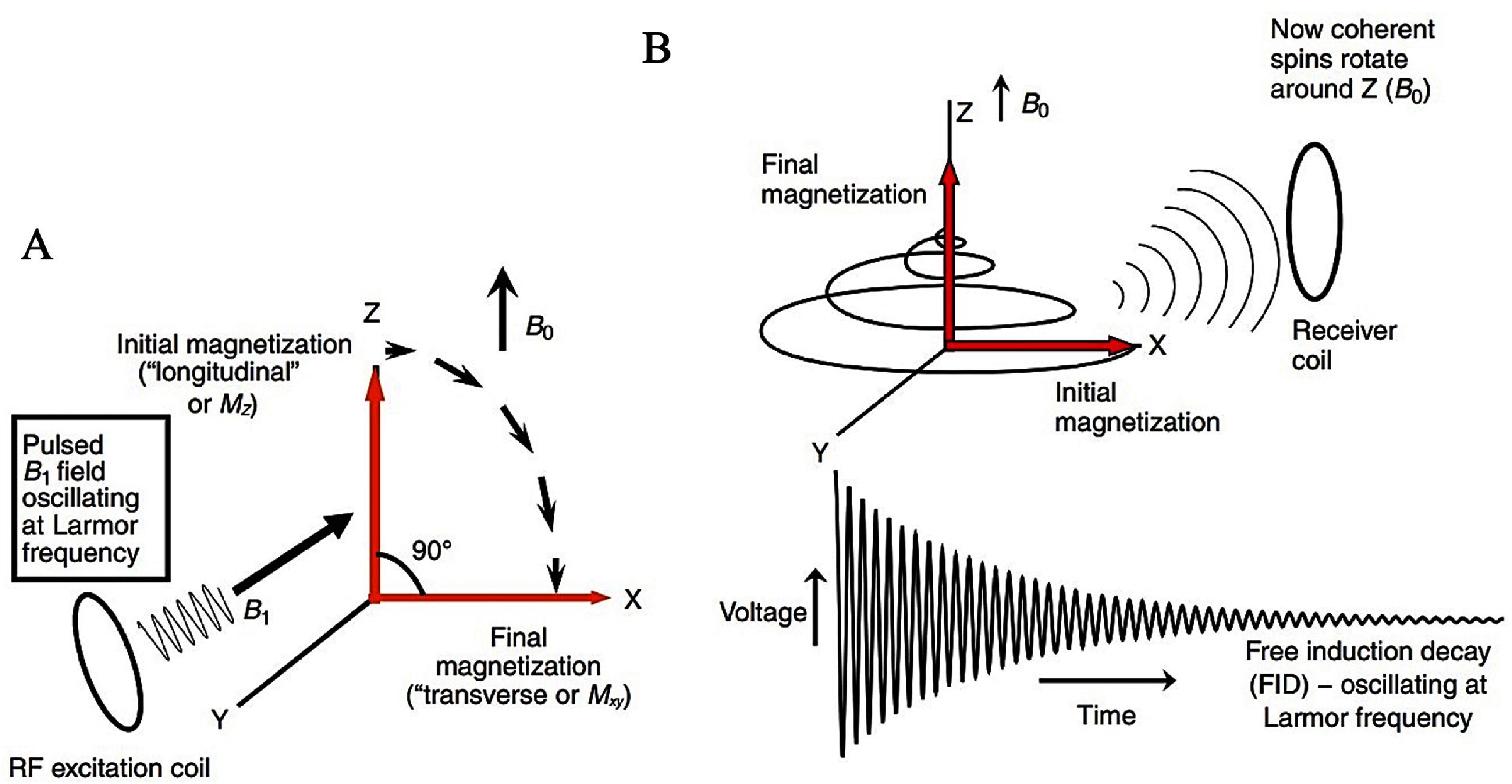
(A) The transformation of longitudinal magnetization (*M_z_*) into transverse magnetization (*M_xy_*) through a 90° RF pulse is depicted. In the rotating frame, the *B*₁ field appears static (along the *Y* axis), causing the magnetization to rotate about the *Y* axis (B) After the *B*_1_ field is taken away, in the laboratory frame, the spins continue to revolve at the Larmor frequency around the *Z* axis, but the signal degrades (in the *X–Y* plane) as equilibrium is restored. As a result, a coherent, oscillating magnetic field is detected by a receiver coil—known as the free induction decay (FID). The frequency of the FID, which is an exponentially decaying sinusoidal voltage signal, is the same as the spins’ Larmor frequency. Reprinted from [42], Copyright (2019), with permission from Elsevier.

In MRI, the signal arises mainly from hydrogen nuclei in tissue water and lipids, which are present at high concentration in most soft tissues [36, 38]. Their nuclear spins carry magnetic moments that respond to the applied magnetic fields and form the physical basis of NMR and MRI signal formation [35, 36, 39]. In the absence of *B*_0_, thermal motion keeps the individual moments randomly oriented, and the net magnetization is effectively zero [35]. However, when the body is placed in the strong static magnetic field *B*_0_ of the MRI scanner, proton magnetic moments tend to align with or against the field and precess around the *B*_0_ axis at the Larmor frequency, *ω*_0_ = *γB*_0_, where *γ* is the gyromagnetic ratio of the proton [39–41]. The longitudinal direction, often known as the *Z*-axis direction, is the direction of the main magnetic field, *B*_0_. The spins produce a net magnetic field along the *Z* axis, which is parallel to the *B*_0_ field. In contrast, the *X–Y* plane, also called the transverse plane, shows no net magnetization because the spins precess randomly around the *Z*-axis. To obtain a measurable signal, the longitudinal magnetization must be flipped into the transverse plane. This is achieved by applying a short RF excitation pulse *B*_1_ at the Larmor frequency using an RF transmit coil. The RF pulse rotates the net magnetization away from the *Z*-axis, creating a coherent transverse component *M_xy_* that precesses in the *X–Y* plane and induces an oscillating voltage in a nearby receiver coil tuned to the same frequency (figures 2(A) and (B)) [28, 42, 43]. By combining RF excitation with magnetic field gradients, only spins within a desired slice are excited, and their spatial positions are encoded in the phase and frequency of the measured signal [41–45]. In simple terms, the MR signal detected by the receive coil is the summed contribution of all precessing spins in the excited volume, whose local resonance frequencies are slightly shifted by the applied gradients. These controlled frequency and phase shifts map spatial information into *k*-space, from which the final image is reconstructed by Fourier transformation [46].

Once the RF pulse is turned off, the magnetization relaxes back toward equilibrium through two key processes. *T*_1_ (longitudinal) relaxation describes the recovery of *M_z_* along the *Z*-axis as spins exchange energy with their surroundings; the *T*_1_ time is the time required for the longitudinal magnetization to reach about 63% of its equilibrium value. However, *T*_2_ (transverse) relaxation describes the decay of *M_xy_* in the *X–Y* plane due to dephasing caused by local magnetic-field variations and spin–spin interactions; the *T*_2_ time is defined as the time for the transverse magnetization to fall to about 37% of its initial value [47, 48]. Both *T*_1_ and *T*_2_ relaxation times are critical in MRI, as they affect image contrast and help differentiate between tissue types. Differences in *T*_1_ and *T*_2_ among tissues are the basis of conventional *T*_1_- and *T*_2_-weighted MRI contrast. For the purposes of this review, the crucial point is that exogenous contrast agents, including gadolinium chelates and MNPs, act by selectively modifying these relaxation processes. Paramagnetic agents predominantly shorten *T*_1_, increasing signal in *T*_1_-weighted images, whereas superparamagnetic nanoparticles mainly enhance dephasing and shorten *T*_2_, producing signal loss in *T*_2_- and *T*_2_*-weighted images. These mechanisms and their implications for the design of MNP contrast agents are explored in the following subsections.

### 2.3. MNPs as contrast agents for MRI

**Figure 3.**
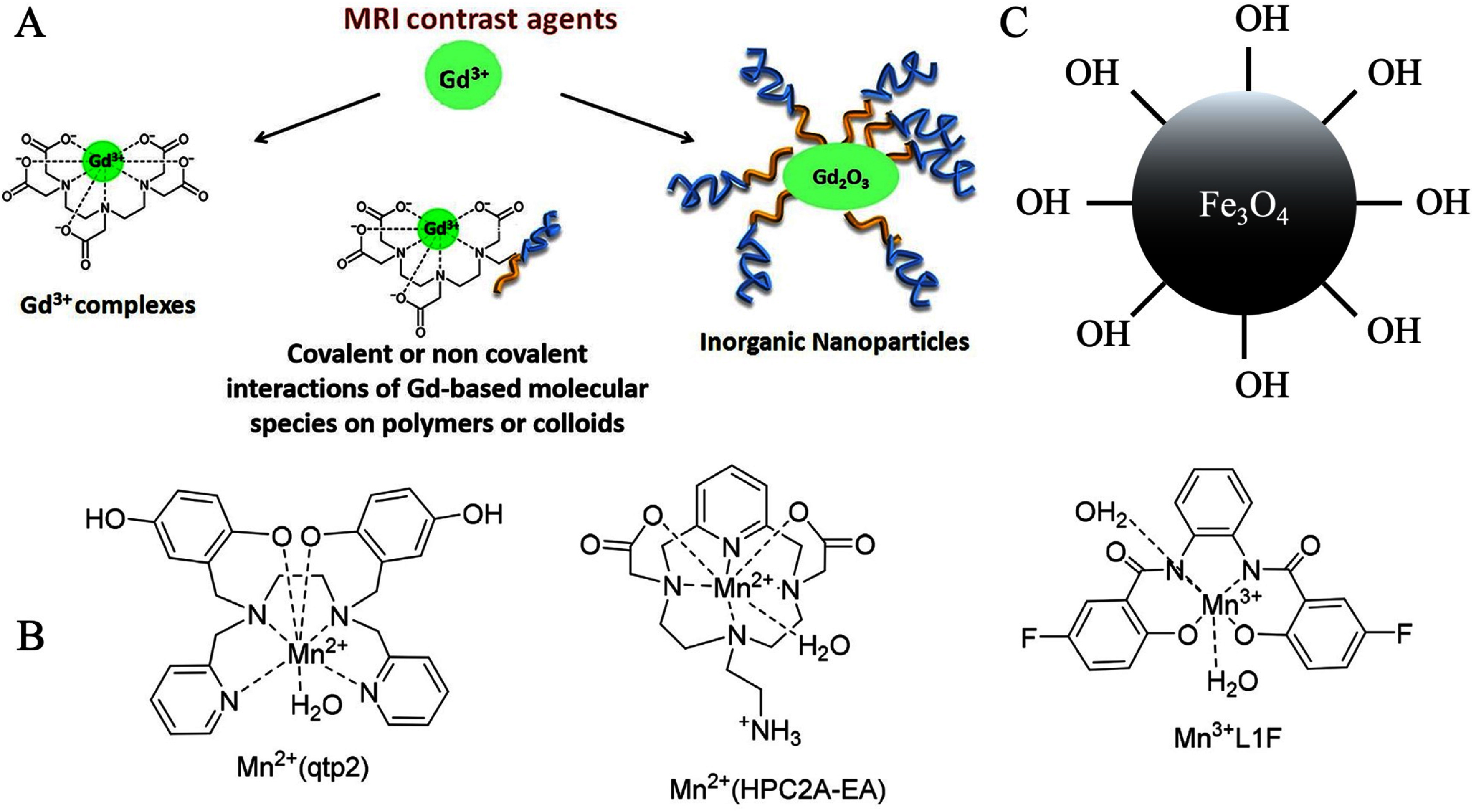
The schematic of the most widely studied MRI contrast agents. (A) Gd-based contrast agent, (B) Mn-based contrast agent, and (C) iron oxide MNPs-based contrast agent. (A) Reprinted from [53], Copyright (2019), with permission from Elsevier. (B) Reprinted from [54], Copyright (2021), with permission from Elsevier. (C) Is an original figure prepared by the authors.

The contrast in MR images is primarily influenced by proton spin density and the longitudinal (*T*_1_) and transverse (*T*_2_) relaxation times. In healthy soft tissues, local differences in relaxation times are often sufficient to generate strong contrast between organs in *T*_1_-weighted and *T*_2_-weighted images. Pathological tissue can also be differentiated from healthy tissue due to variations in *T*_1_ and *T*_2_ relaxation times. However, many clinical conditions do not produce distinct changes in relaxation times or noticeable morphological alterations. In such cases, MRI contrast agents are used to modify the relaxation times of affected tissues, thereby enhancing the visibility of pathological changes. Figures 3(A)–(C) categorizes different classes of MRI contrast agents, with a focus on those highlighted in this review, including gadolinium-based agents (figure 3(A)), manganese-based agents (figure 3(B)), and SPIONs (figure 3(C)) [25, 49, 50]. This opens a wide range of MRI applications for therapeutic medicine in addition to diagnostic radiology [50, 51]. Contrast agents enhance MRI imaging of tissues and organs by altering water proton relaxation times, thereby increasing the signal intensity in the regions where they accumulate. So, targeted administration of contrast agents into tumor tissues is essential. Tumors have leaking blood vessels and differ physiologically from healthy tissue. This means that the tumor vascular permeability to macromolecules allows nanoscale contrast agents to passively accumulate in the tumor. Numerous cancer-related biomarkers that can be targeted for molecular imaging are also expressed by tumor tissues on the extracellular matrix, tumor cell surface, or angiogenic microvessels. Designing targeted contrast agents requires two key components: highly binding ligands and abundantly expressed cancer biomarkers. Together, they produce sufficient contrast for visualizing these biomarkers with CE MRI [52].

**Figure 4.**
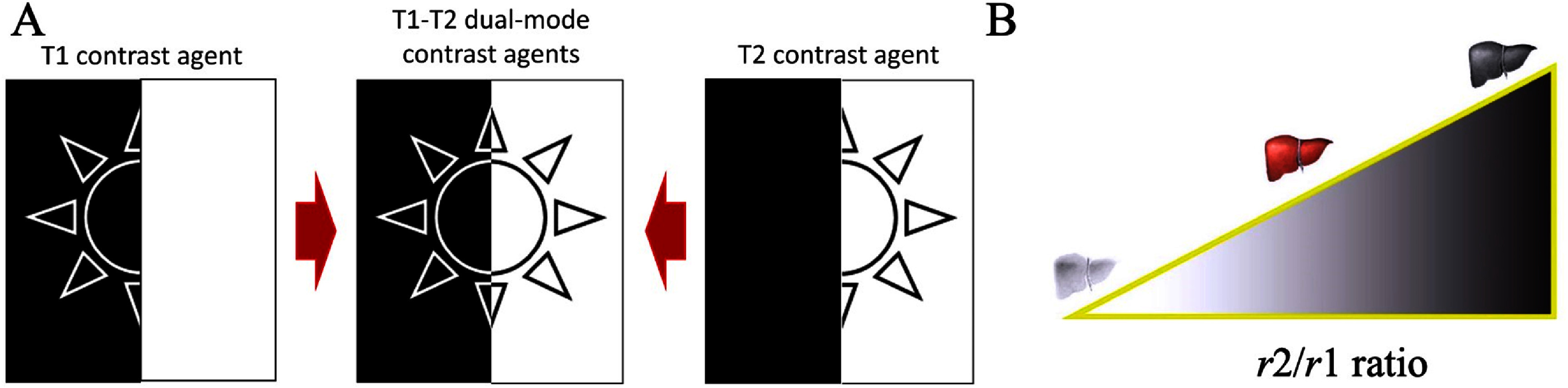
(A) Schematic representation of *T*_1_, *T*_2_, and *T*_1_–*T*_2_ dual contrast effects. (B) Influence of the *r*_2_/*r*_1_ ratio on the efficiency of a contrast agent. (A) Reprinted from [59], Copyright (2021), with permission from Elsevier. (B) [60] (2015), reprinted by permission of the publisher (Taylor & Francis Ltd, www.tandfonline.com).

The rates of all the relaxation processes are typically sped up by an MRI contrast agent, but only one of them is primarily affected. Positive or *T*_1_ contrast agents are those that primarily shorten the relaxation time of the longitudinal component of the magnetization, whereas negative or *T*_2_ contrast agents primarily shorten the relaxation time of the transverse component [45, 55, 56]. It is also important to know that the effectiveness of a contrast agent is typically measured using longitudinal relaxivity (*r*_1_), transverse relaxivity (*r*_2_), and the relaxivity ratio (*r*_2_/*r*_1_). Generally, a contrast agent with a low *r*_2_/*r*_1_ ratio is categorized as a *T*_1_ agent, providing bright contrast in images, while an agent with a higher *r*_2_/*r*_1_ ratio is classified as a *T*_2_ agent, resulting in darker (negative) contrast. Since the relaxation rates *R*_1_ and *R*_2_ are the reciprocals of the relaxation times *T*_1_ and *T*_2_ (i.e. *R*_1_ = 1/*T*_1_, *R*_2_ = 1/*T*_2_), an agent with high *r*_1_ effectively reduces *T*_1_, enhancing signal intensity, whereas an agent with high *r*_2_ shortens *T*_2_, leading to signal loss. This classification is illustrated in figure 4(B) [55, 57, 58].

However, through self-confirmation and improved discrimination between healthy and diseased organs and tissues, the combination of *T*_1_ and *T*_2_ contrast effects in a single contrast agent can produce more accurate MRI results, as depicted in figure 4(A). Multiple imaging modalities offer synergistic advantages over single-modality approaches, providing complementary diagnostic insights. The *T*_1_–*T*_2_ dual-modal MRI technique has attracted considerable attention due to its capability to deliver precise diagnostic information, leveraging the beneficial contrast effects of *T*_1_ imaging for high tissue resolution and *T*_2_ imaging for effective lesion detection. Additionally, multimodal imaging within a single device is highly advantageous, as it accommodates the varied penetration depths and spatial/temporal resolutions of different imaging methods. There is a critical need for advanced dual-modal contrast agents to enhance the effectiveness of *T*_1_–*T*_2_ dual-modal MRI techniques [59, 61–63].

#### 2.3.1. T_1_ contrast agents

**Figure 5.**
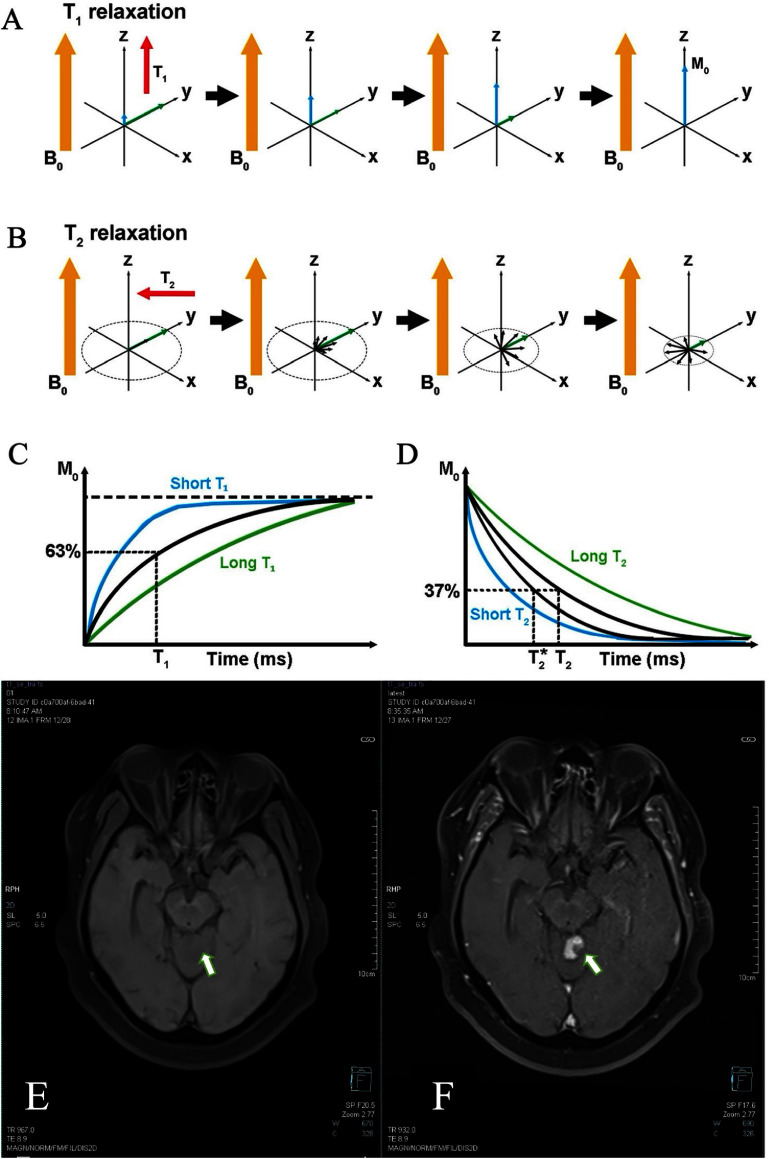
(A) and (B) are schematic illustrations of the fundamental ideas of MRI. (C) *T*_1_ recovery curve. (D) *T*_2_ decay curve. *T*_1_-weighted MR images of the brain acquired before (E) and after (F) Gd-based contrast administration. After contrast injection, a strongly enhancing lesion becomes visible in the cerebellar vermis (short arrow in D), measuring approximately 1.8 × 1.5 cm. (A)–(D) Reproduced from [71]. CC BY 4.0. (E) and (F) Reprinted from [72], Copyright (2024), with permission from Elsevier.

*T*_1_ relaxation times vary across biological systems, influencing the contrast in *T*_1_-weighted MRI. In *T*_1_-weighted imaging, fat generally appears brighter than water due to differences in chemical composition and molecular motion dynamics. *T*_1_ contrast agents further enhance these variations by selectively shortening *T*_1_ relaxation times in specific tissues, improving overall image contrast (for example, pre- and post-contrast *T*_1_-weighted brain images in figures 5(E) and (F) show signal enhancement in lesions after administration of a *T*_1_ agent). Paramagnetic nanoparticles have been produced as novel *T*_1_ contrast agents due to low magnetic anisotropy (small *r*_2_) and a large paramagnetic characteristic (large *r*_1_). Nanoparticles made of various transition and lanthanide metal complexes may make excellent *T*_1_ MRI contrast agents because they have a lot of metal ions with high magnetic moments on their surface, and the presence of these in organs or tissues may shorten the *T*_1_–relaxation time (figures 5(A) and (C)) [64]. These paramagnetic contrast agents enhance proton relaxation through interactions with the protons that accelerate the return to equilibrium. Unpaired electrons of paramagnetic ions create fluctuating magnetic fields that interact with nearby proton spins through dipole–dipole interactions, facilitating energy transfer and increasing the rate of relaxation [65]. The interactions between protons and paramagnetic ions can occur in two places: the inner and outer spheres. Inner sphere relaxation involves water molecules directly interacting with the paramagnetic center. Outer sphere relaxation, in contrast, occurs through interactions with water molecules diffusing near the contrast agent, generating weaker dipole interactions compared to the ones in the inner sphere. This process depends on key factors such as molecular size, hydration number of the contrast agent, electron spin relaxation time, and correlation time of molecular motion, which govern how efficiently the energy is exchanged [65]. The work of Bloembergen, Purcell, and Pound established the theoretical framework for understanding nuclear relaxation, highlighting the importance of dipolar interactions between nuclear and electronic spins in paramagnetic environments [66]. Their work led to a set of equations that allow a detailed correlation between paramagnetic relaxation enhancements and molecular properties [67–70]. This theoretical integration serves as a critical tool for interpreting the performance of contrast agents in MRI, particularly those involving gadolinium (Gd)-based systems.

Among all the paramagnetic nanoparticles, Gd-based compounds are considered the most important and widely used *T*_1_-weighted MRI contrast agents. This prominence is attributed to the gadolinium ions (Gd^3+^), which possess seven unpaired electrons, providing a high magnetic moment that significantly enhances the *T*_1_ relaxation of nearby water protons. Additionally, Gd^3+^ is particularly favored for creating Gd-chelates due to its greater number of unpaired electrons with parallel spin compared to other paramagnetic ions, making it highly effective for MRI contrast enhancement [73]. Gd-based *T*_1_-weighted MRI contrast agents exist in various forms, including small-molecule chelates, nanoparticles, macromolecule conjugates, and hybrid systems. Small-molecule chelates, such as Magnevist (Gd-DTPA), Dotarem (Gd-DOTA), and Gadobenate (Gd-BOPTA), are clinically approved agents that provide rapid contrast enhancement but have fast renal clearance [74]. Gd-based nanoparticles, such as Gd_2_O_3_, GdF_3_, NaGdF_4_, and KGdF_4_, have been widely studied for MRI applications. Macromolecule-conjugated Gd contrast agents, including Gd-liposomes, polymeric Gd, and albumin-bound Gd, enable functionalization and targeting [52, 75, 76]. These diverse formulations allow for enhanced contrast efficiency, improved biodistribution, and optimized imaging performance in MRI [77]. Although Gd-based contrast agents (GBCAs) effectively shorten the *T*_1_ relaxation time and enhance image brightness in ROI [25, 78, 79], concerns over their association NSF have prompted researchers to explore alternative contrast agents [80]. To address this issue, scientists have been actively investigating safer alternatives that retain strong *T*_1_ relaxation properties while reducing potential NSF risks. Among these alternatives, manganese (Mn)-based complexes, such as MnO, have emerged as promising candidates for *T*_1_ MRI contrast imaging [81, 82].

#### 2.3.2. T_2_ contrast agents

Another class of MR contrast agents is superparamagnetic nanoparticles that have been primarily used as negative (*T*_2_) contrast agents and are typically iron oxide nanoparticles (see figure 5(E) as an example). They are anticipated to induce negative contrast (*T*_2_) effects by decreasing the relaxation times at places where they are accumulated after intravenous injection (figures 5(B) and (D)) [83, 84]. Due to their favorable magnetic characteristics and biocompatibility, magnetite (Fe_3_O_4_) and maghemite (*γ*-Fe_2_O_3_) nanoparticles with sizes around 20 nm have been used more frequently for bioimaging. The NMR transverse relaxation rate (*R*_2_) of the water protons increases when iron oxide nanoparticles with high magnetic susceptibility are exposed to an external magnetic field, because they produce strong local field inhomogeneities. *T*_2_ relaxation generally occurs at the same time as *T*_1_ relaxation, but it can also take place independently. This occurs when a spin experiences a localized disturbance in the static magnetic field in addition to the primary magnetic field [70, 85]. For instance, a spin of a water molecule near an iron-containing particle may experience such a disturbance. When the disturbance in the field aligns with the static field, they combine, causing the spins to precess at a faster frequency, while unaffected spins precess at the original Larmor frequency. As time passes, a phase difference develops between the altered spin and the other spins. This phase difference leads to a decrease in net transverse magnetization, causing *T*_2_ relaxation [33, 34, 86, 87]. The transverse relaxivity (*r*_2_), which is the proton relaxation rate enhancement per millimolar iron and is a function of nanoparticle size, magnetic properties, and aggregation, is used to express the performance of the contrast agent. In *T*_2_-weighted MRI, higher transverse relaxivities correspond to improved image contrast [88].

Due to their nanoparticulate structures, nanoparticle-based contrast agents have several benefits over conventional paramagnetic agents. The agents have large specific surface areas that make conjugation with targeting molecules and other probes for the creation of targeting and multimodal agents easier. However, while surface functionalization of nanoparticles allows for versatile ligand attachment, the stability of these conjugations depends on the binding mechanism [89]. In addition, the agents’ magnetic properties can be tailored by altering their size, shape, composition, and assembly. Also, the agents’ nanoscale size, adaptable surface structure, and shape enable variable and advantageous biodistribution [90]. Thus, nanoparticles are frequently used for targeted molecular MRI in several research fields, such as cancer detection and monitoring of treatment, e.g. antiangiogenic and proapoptotic, and diseases with an inflammatory or cell death complication [83, 84]. However, particle size, toxicity, and surface properties such as hydrophilicity and surface charge of nanoparticles significantly impact opsonization, which in turn affects the clearance rate and tissue distribution of intravenously injected MNPs. For MNP suspensions, the pH should ideally be near 7.4, and the MNP surfaces should be hydrophilic, as hydrophobic particles are more rapidly opsonized and cleared, impacting tumor penetration but leading to quicker clearance through renal filtration and extravasation [91].

A key challenge in developing MNPs for effective clinical use is the fine-tuning of surface coating materials, alongside selecting an appropriate magnetic core. The coating can involve long-chain organic ligands or inorganic/organic polymers, introduced either during synthesis (*in situ*) or after synthesis (post-synthetic). There are two primary approaches for attaching surface molecules to the magnetic cores: end-grafting and surface encapsulation. In end-grafting, a single capping group at one end of the coating molecule anchors it to the magnetic core. In contrast, surface encapsulation uses polymers with multiple active groups that form several bonds to the core, resulting in a more robust and stable coating [92, 93].

#### 2.3.3. Dual T_1_/T_2_ contrast agents

Conventional MRI contrast agents are generally effective in either *T*_1_ or *T*_2_ imaging modes alone, which can sometimes lead to diagnostic uncertainties, especially when targeting small biological targets. The development of a contrast agent that simultaneously provides strong *T*_1_ and *T*_2_ effects could represent a breakthrough, as it offers the potential for enhanced accuracy through self-confirmation, allowing for clearer differentiation between healthy and diseased tissues [60, 94]. Thus, the creation of multimodal imaging probes is becoming increasingly popular. By enhancing anatomical details in the MR images, dual-mode *T*_1_–*T*_2_ contrast agents, which combine the benefits of positive and negative contrasts, may enable better diagnosis [23, 51, 60]. In *T*_2_-weighted MRI produced by iron oxide nanoparticles, the resulting dark signal can sometimes confuse the clinical diagnosis because it can be confused with signals from bleeding, calcification, or metal deposits, and susceptibility artifacts distort the background image [95–97]. Therefore, there is an urgent need for the creation of novel classes of nanoparticulate MR contrast agents with either *T*_1_ or *T*_1_/*T*_2_ dual-contrast capability [90, 94, 98]. The effectiveness of an MRI contrast agent as a *T*_1_ or *T*_2_ agent is influenced by several key parameters, including metal ion composition, nanoparticle size, surface properties, and aggregation state. Metal ion selection plays a crucial role, as paramagnetic ions like Gd^3+^ and Mn^2+^ enhance *T*_1_ contrast, while SPIONs and metallic nanoparticles induce strong *T*_2_ effects. Nanoparticle size impacts relaxivity, where smaller particles (2–5 nm) favor *T*₁ contrast due to better water accessibility, whereas larger particles (>10 nm) enhance *T*_2_ relaxivity, by increasing local magnetic field inhomogeneities. Surface coating and functionalization also influence water diffusion near the nanoparticle core; hydrophilic coatings (e.g. PEG, citrate) improve *T*_1_ relaxivity, while hydrophobic or clustered coatings (e.g. dextran, micelles) enhance *T*_2_ contrast by restricting water accessibility. Additionally, nanoparticle aggregation can further amplify *T*_2_ effects by creating stronger magnetic dipole interactions, while well-dispersed particles favor *T*_1_ contrast. Also, as magnetic field strength (*B*_0_) increases, *r*_1_ relaxivity generally decreases, while *r*_2_ increases, resulting in a higher *r*_2_/*r*_1_ ratio. Moreover, the linear dependence of 1/*T*_2_ on *B*_0_ suggests that *T*_2_ effects become more dominant at stronger fields, which can overshadow *T*_1_ contrast if not carefully optimized. To ensure effective dual-modal imaging, contrast agents must be designed with balanced relaxivity profiles, preventing excessive *T*_2_-induced signal loss while maintaining sufficient *T*_1_ enhancement [99, 100]. Lastly, magnetic anisotropy, saturation magnetization (*M*_s_), and electronic spin relaxation time affect the *r*_2_/*r*_1_ ratio, determining whether a contrast agent exhibits a dominant *T*_1_ or *T*_2_ effect in MRI imaging applications [101]. For dual-contrast agents, it is crucial to carefully regulate the *r*_2_ of MNPs to prevent excessive *T*_2_ decay, which can overshadow *T*₁ relaxation and reduce overall contrast enhancement. Strong *T*_2_ decay may diminish the *T*₁ signal, impairing dual-modal imaging performance [51]. However, dual-modal *T*_1_–*T*_2_ MRI contrast agents should exhibit both high *r*_1_ and *r*_2_ relaxivities, with *r*_2_/*r*_1_ ratios (∼2–10) falling between those of optimal *T*_1_ and *T*_2_ contrast agents. For MNP-based dual modal contrast agents, their typically low *r*₂/*r*₁ ratios require a reduction in their inherently high *r*_2_/*r*_1_ ratios [102].

## 3. MRI in biomedical imaging applications

Medical imaging is a cutting-edge technology that reveals intricate details of how the human body functions. In the field of medical imaging technology, MRI is widely used to diagnose a variety of problems in different parts of the body. In this section, we will dig into various MRI imaging areas, such as cardiac, breast, brain, and musculoskeletal imaging, each of which plays a crucial part in understanding and treating particular medical disorders.

### 3.1. Cardiac MRI

CMR has been used more frequently over the past 10 yr to assess cardiovascular disease and has replaced other imaging modalities for patients with many congenital and acquired cardiovascular disorders, such as congenital heart disease, MI, dilated cardiomyopathy, and large-vessel disease [103, 104]. In addition, CMR can assist in the diagnosis of a complex physiological condition known as heart failure, which is defined by a decreased cardiac output that is insufficient to meet systemic demands. CMR is of paramount importance in clinical diagnosis since most deaths worldwide are caused by cardiovascular diseases, which account for around 30% of all deaths [105–108]. Additionally, primary cardiac tumors, secondary or metastatic cardiac tumors, intracavitary thrombus, or cardiac pseudotumors are several types of heart masses that can be detected via CMR. From these tumors, metastatic masses are around 40 times more common than primary heart tumors, and initial cardiac tumors are uncommon and normally benign [104]. CMR enables precise tissue characterization and localization of cardiac masses, as well as the ability to assess the mass’s functional impact and degree of involvement. Due to the vast range of underlying etiologic processes that can affect the heart, such as ischemic heart disease, myocarditis, basic myocardial disease, hypertension, valvular heart disease, and acquired infiltrative and pericardial illnesses, the pathophysiology of this syndrome is complex. The proper prescription of care for this population depends critically on the distinction of these disorders. Current recommendations for treating heart failure identify three general evaluation steps: (1) characterizing the myocardial and valvular structures and their function; (2) differentiating between ischemic and nonischemic causes, including identifying substrates that may be modifiable; and (3) risk stratification for therapeutic management, such as the benefit of revascularization [109]. All three of these crucial processes may be addressed with the help of contemporary CMR techniques, which consolidate diagnostic testing into a single imaging technique. Monitoring of congestive heart failure patients is possible with the measurement of their cardiac morphology, function, flow, perfusion, tissue injury, and fibrosis in a single imaging test with the help of specific SE pulse sequences [107, 109].

**Figure 6.**
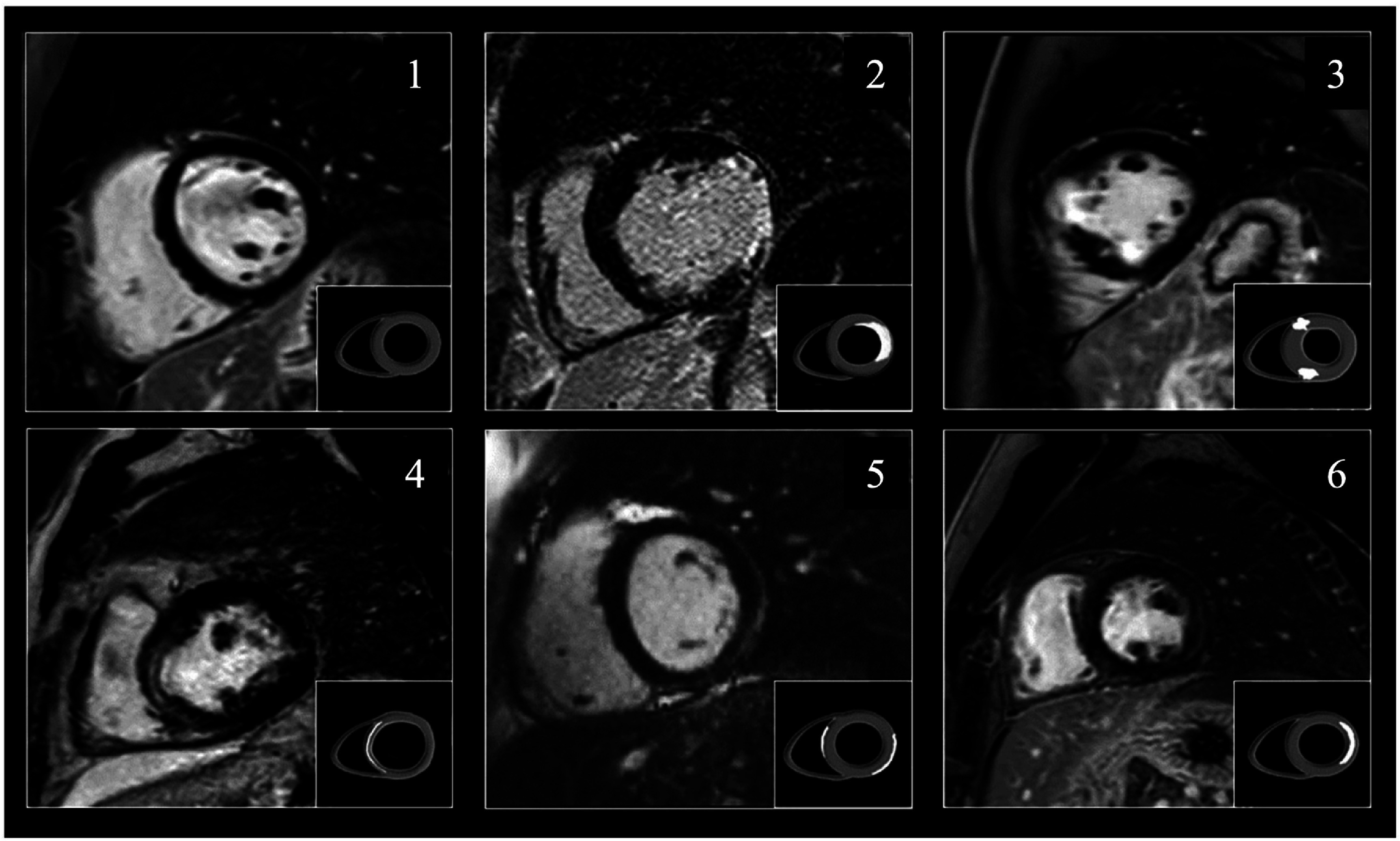
Late gadolinium-enhanced cardiovascular MRI (short-axis views of the left ventricle) illustrates typical patterns of myocardial contrast uptake in different diseases. In all panels, the dark circular region in the center is the blood pool, and the surrounding ring is the left-ventricular myocardium. Bright (white) areas in the myocardial wall indicate regions where gadolinium contrast accumulates because of infarction or fibrosis. (1) Normal myocardium: the wall appears uniformly dark, with no late enhancement. (2) Ischemic cardiomyopathy: bright enhancement starting at the inner (subendocardial) layer of the wall, following a coronary artery territory, consistent with myocardial infarction. (3, 4) Hypertrophic cardiomyopathy: patchy mid-wall enhancement in thickened myocardium, reflecting focal fibrosis. (5) Fabry disease: characteristic enhancement of the inferolateral wall. (6) Myocarditis: subepicardial or mid-wall enhancement in the lateral wall, typical of inflammatory injury. Reprinted from [110], Copyright (2019), with permission from Elsevier.

In CMR, Specific SE sequences have been created to image cardiovascular structures. Suppressing the signal from the blood and fat is important to highlight the myocardial or vascular wall signal. To achieve this, the signal from the blood is suppressed using inversion RF preparation pulses, giving the image a ‘dark blood’ appearance. To suppress the signal from the fat, additional RF pulse saturation or inversion may be added, giving the appearance of a ‘dark blood and dark fat’ image [107]. These non-contrast SE techniques provide high-resolution anatomy and wall structure, but tissue characterization is further improved by CE imaging. At present, clinically used CMR contrast agents are almost exclusively extracellular Gd^3+^-based chelates, which are employed in late gadolinium enhancement (LGE) protocols. In LGE imaging, regions with expanded extracellular space due to necrosis or fibrosis retain more gadolinium and therefore appear bright (hyperenhanced) in the left-ventricular wall. The pattern and location of this hyperenhancement are used to distinguish normal myocardium from ischemic scar and non-ischemic cardiomyopathies such as hypertrophic cardiomyopathy, Fabry disease, and myocarditis in MI patients (as shown in figure 6).

**Figure 7.**
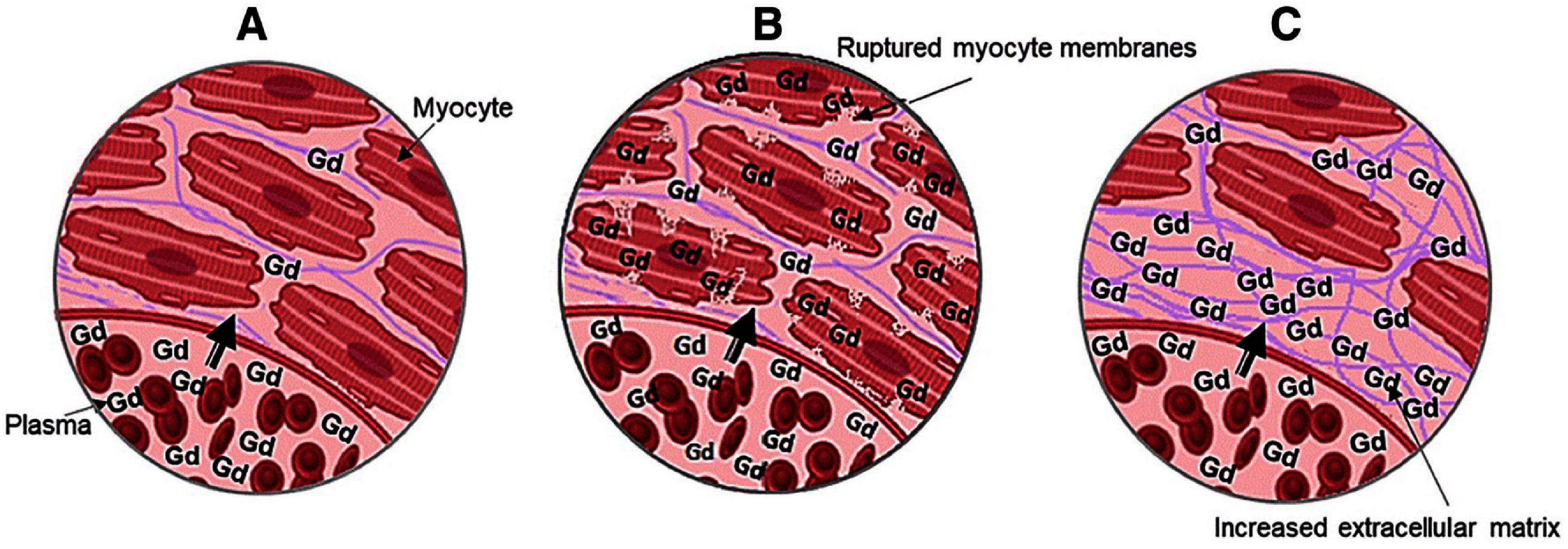
Conventional Gd-based contrast agents’ working mechanism. (A) In normal myocardium, there is no Gd enhancement. (B) In an acute infarct, Gd enters ruptured cell membranes and causes enhancement. (C) In a chronic infarct, there is Gd enhancement due to increased extracellular space and fibrotic scar deposition. Reprinted from [110], Copyright (2019), with permission from Elsevier.

Conventional GBCAs have a nonspecific extracellular distribution due to their low molecular weight. They can rapidly extravasate from the intravascular space but do not cross intact myocyte cell membranes, so in normal myocardium, both the distribution volume and the total amount of Gd within the tissue are small, and no late enhancement is seen (figure 7(A)). In acute infarction, myocyte membranes rupture, allowing Gd to diffuse into the intracellular space; the enlarged distribution volume and higher local concentration of Gd produce a bright hyperenhanced region on CMR (figure 7(B)). In chronic infarction or cardiomyopathy, myocytes are replaced by collagenous/fibrotic scar, and the interstitial (extracellular) space is expanded, leading again to increased Gd accumulation and persistent hyperenhancement in those regions (figure 7(C)) [110].

**Figure 8.**
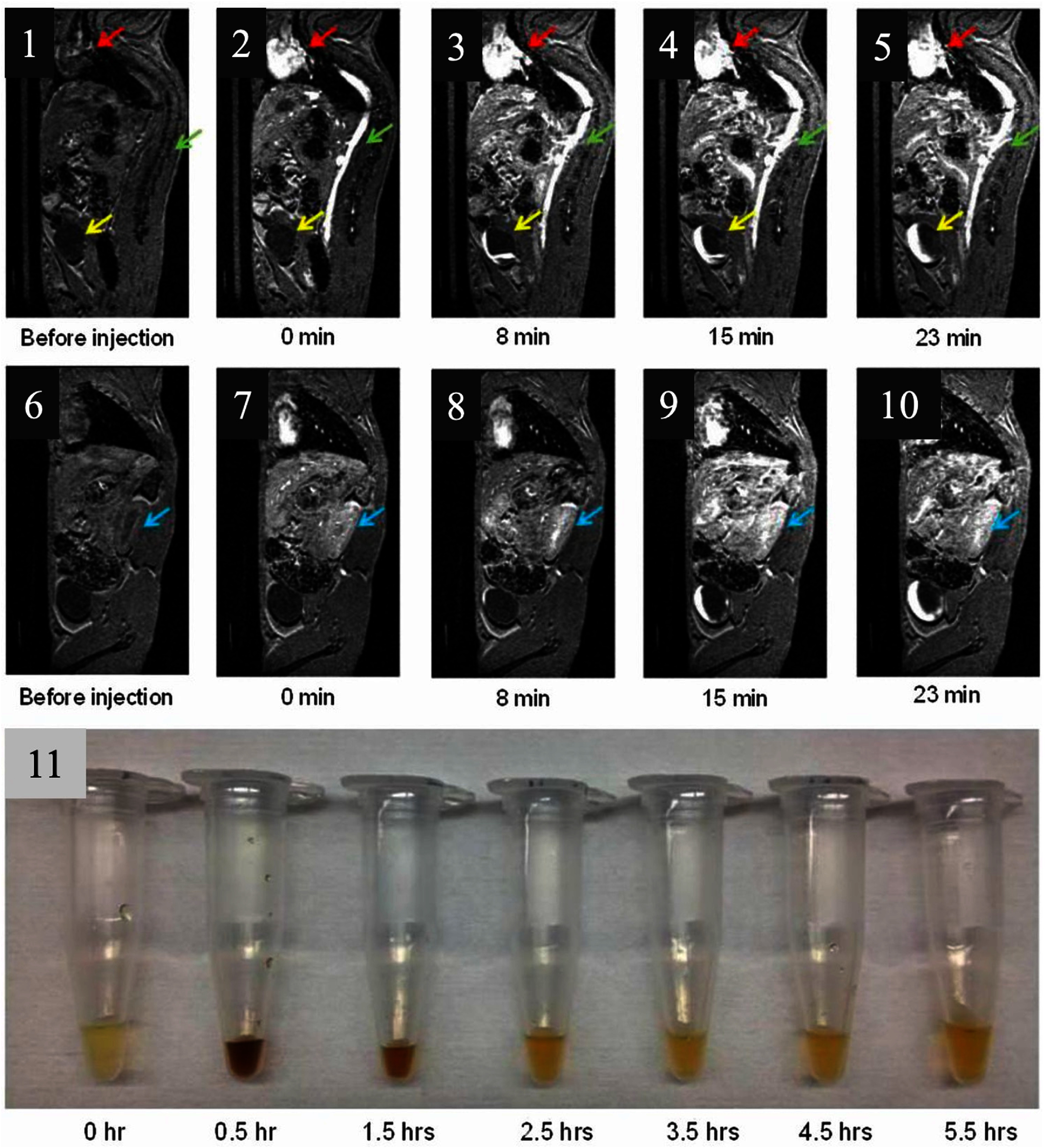
Serial *T*_1_-weighted MRI demonstrates blood-pool enhancement followed by renal filtration and urinary excretion of Zwitterionic dopamine sulfonate (ZDS)-coated ultrasmall SPIONs (ZES-SPIONs). (1–5) Serial *T*_1_-weighted sagittal MR images of a mouse before injection and at 0, 8, 15, and 23 min after intravenous administration of ZES-SPIONs acquired at 7 T. Immediately after injection (0 min), blood-pool signal becomes brighter (positive *T*_1_ contrast) in the heart (red arrow) and vena cava (green arrow), consistent with vascular enhancement. By 8 min, the bladder (yellow arrow) begins to brighten, with progressive intensification at 15–23 min, indicating urinary excretion. (6–10) A second sagittal slice at the same time points highlights the kidney (blue arrow), showing increasing enhancement after injection consistent with renal filtration. (11) Corresponding urine samples collected at 0, 0.5, 1.5, 2.5, 3.5, 4.5, and 5.5 h post-injection show transient darkening at early time points (0.5–1.5 h), visually supporting urinary elimination and rapid renal clearance *in vivo*. Reproduced with permission from [111].

Wei *et al* reported zwitterion (ZDS)-coated exceptionally small SPIONs (ZES-SPIONs) for *T*_1_-weighted CE MR imaging and angiography in mice and rats [111]. SPIONs were synthesized from the thermal decomposition of Fe(oleate)3 in the presence of oleic acid. The ZES-SPIONs were injected intraperitoneally (IV) into mice, and urine was collected at various intervals to further visualize renal clearance. Mice received an injection of ZES-SPIONs at a concentration of 0.2 mmol [Fe]/kg and were then imaged using an MRI scanner. This concentration was similar to that of GBCAs (∼0.1–0.25 mmol [Gd]/kg) used in traditional MRI. One of the mice’s *T*_1_-weighted MR images can be found in figures 8(1)–(10). Normal urine color before injection is depicted in figure 8(11). At 30 min and 1.5 h post-injection, the urine became dark brown, which is characteristic of ZES-SPION elution. As anticipated, the sagittal slice shown in figure 8(1), before the injection of ZES-SPIONs, shows no discernible positive contrast in the heart (red arrow), vena cava (green arrow), or bladder (yellow arrow). Figure 8(2) shows a considerable rise in positive contrast in the vena cava and heart after injection. The bladder starts to show positive contrast at 8 min after injection (figure 8(3)), signifying the excretion of ZES-SPIONs. Figures 8(4) and (5) demonstrate how the contrast in the bladder intensifies with time, indicating a progressive buildup of ZES-SPIONs. Another sagittal slice taken at the same time points is shown in figures 8(6)–(10). The kidney (blue arrow) exhibits no contrast enhancement before injection (figure 8(6)). Nevertheless, the kidney shows a noticeable rise in positive contrast right after injection. Figures 8(7)–(10) show a persistent increase in positive contrast in the kidney over time. According to these results, ZES-SPIONs are cleared by the kidneys at a pace that is appropriate for multiphase dynamic imaging procedures.

To further illustrate the renal clearance process, they performed biodistribution research utilizing ^59^Fe radioisotope-labeled ZES-SPIONs to assess the *in vivo* stability of the ZES-SPION formulation. Results showed that 65% ± 1.4% of the injected dose was cleared renally within 4 h, as measured by a radioactivity counter. At 24 h post-injection, 13% ± 0.70% remained in the liver, with another 12% distributed across blood, spleen, kidney, lung, gastrointestinal tract, heart, and tail, while 13% remained in the carcass. These results confirm efficient renal clearance of ZES-SPIONs with minimal organ retention.

**Figure 9.**
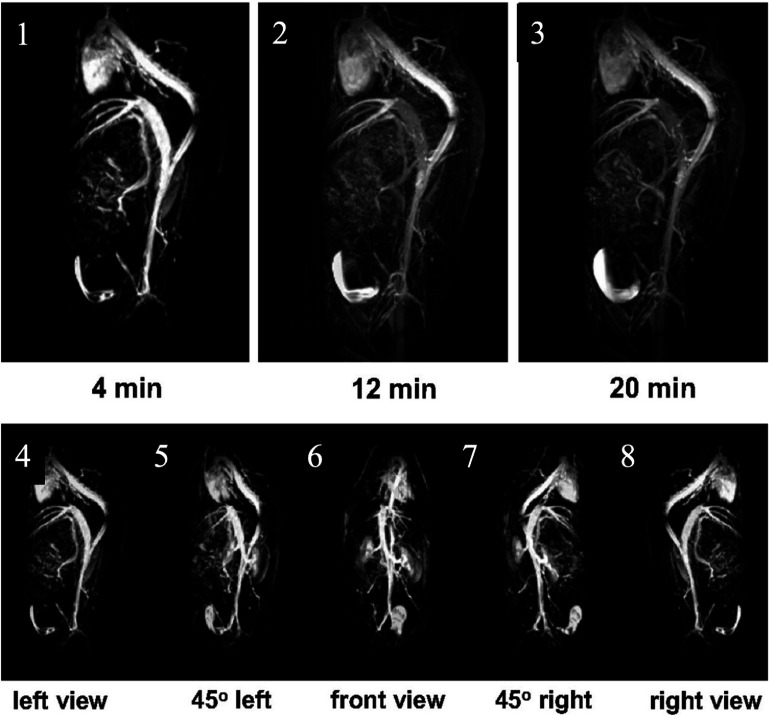
*T*1-weighted MR angiography (MRA) of a mouse after intravenous injection of ZES-SPIONs at 7 T shows early vascular enhancement followed by urinary excretion. (1–3) Maximum-intensity-projection (MIP) images acquired at 4, 12, and 20 min post-injection. Early after injection (4 min), strong blood-pool enhancement is observed in the heart and vena cava, which decreases over time, while signal in the bladder increases, consistent with renal clearance and urinary accumulation. For anatomical reference (heart, vena cava, bladder, and kidney locations), see the arrow annotations in figure 8, which use the same imaging orientation. (4–8) Five different 3D viewing angles (left, 45° left, front, 45° right, right) of the 4 min angiogram, showing the detailed depiction of the arterial tree. Reproduced with permission from [111].

The authors also showed the ZES-SPIONs’ potential for preclinical usage in *T*_1_-weighted MRA, a crucial clinical application for *T*_1_-weighted contrasts. Figure 9 shows 3D angiograms of a mouse at 4, 12, and 20 min after intravenous injection of ZES-SPIONs. At 4 min (figure 9(1)), the heart and arterial tree appear brightly outlined against a dark background, indicating a high concentration of nanoparticles within the blood pool. At later time points (figures 9(2) and (3)), this vascular signal gradually decreases, while a bright signal appears in the bladder, consistent with renal excretion of the particles. Figures 9(4)–(8) displays five different viewing angles of the 4 min angiogram (left, 45° left, frontal, 45° right, and right views), illustrating that a single injection of ZES-SPIONs can provide a detailed 3D map of the major vessels. Pharmacokinetic analysis showed a blood half-life of approximately 19 min, which is much longer than that of the extracellular agent Gd-DTPA (∼2 min) and comparable to the clinical blood-pool agent gadofosveset (Ablavar, ∼23 min). Taken together, these data support ZES-SPIONs as a promising Gd-free candidate for angiographic applications.

**Figure 10.**
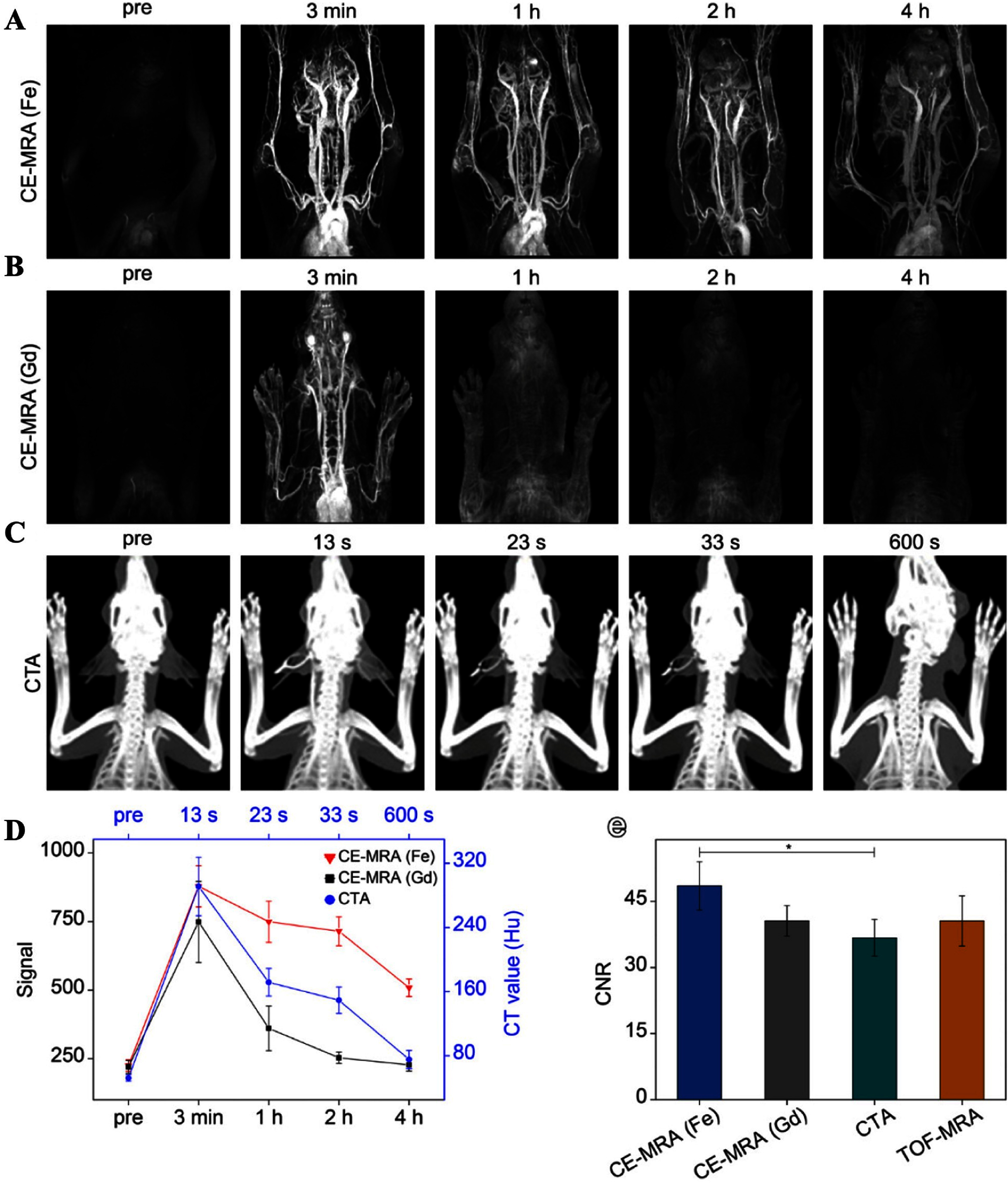
(A) Contrast-enhanced MRA images of normal rabbits acquired before and after intravenous injection of PUSIONPs, and (B) corresponding images obtained with Gd-DTPA at multiple time points. (C) CT angiography images of the same rabbits before and after injection of the iodinated contrast agent Ultravist. (D) Time course of MRI signal/CT value enhancement for PUSIONPs, Gd-DTPA, and Ultravist, showing that the vascular signal produced by PUSIONPs is more prolonged than that of Gd-DTPA or Ultravist. (E) CNR measured in the common carotid artery for USIONPs Gd-based CE-MRA, CTA, and TOF-MRA. Reprinted with permission from [112]. Copyright (2025) American Chemical Society.

More recently, Lu *et al* reported a clinically oriented PEGylated ultrasmall iron oxide nanoparticle formulation (PUSIONPs) as a high-relaxivity blood-pool agent for CE MRA of the carotid and cerebral vasculature [112]. The particles combine a small core size (3.1 nm) with hydrophilic surface engineering, giving a *r*_1_ relaxivity of ∼6.2 mM^−1^ s^−1^ at 3 T and a prolonged intravascular half-life, which together support high signal-to-noise and contrast-to-noise ratios (CNRs) in time-resolved head-and-neck MRA. In rabbit models, CE-MRA with PUSIONPs (figure 10(A)) provided a sharper depiction of carotid and intracranial arteries than conventional Gd-based CE-MRA (figure 10(B)) and iodinated CT angiography (CTA, figure 10(C)). The corresponding time–intensity curves (figure 10(D)) show that the vascular signal generated by PUSIONPs is both stronger and more sustained than that of Gd-DTPA or Ultravist, allowing high-quality angiograms to be obtained up to 4 h after a single injection. Quantitative analysis of CNR in the common carotid artery (figure 10(E)) further demonstrates that PUSIONP-enhanced CE-MRA achieves equal or higher CNR than Gd-based CE-MRA, CTA, and non-contrast TOF-MRA, supporting ultrasmall iron-oxide blood-pool agents as realistic Gd-free alternatives for vascular imaging.

**Figure 11.**
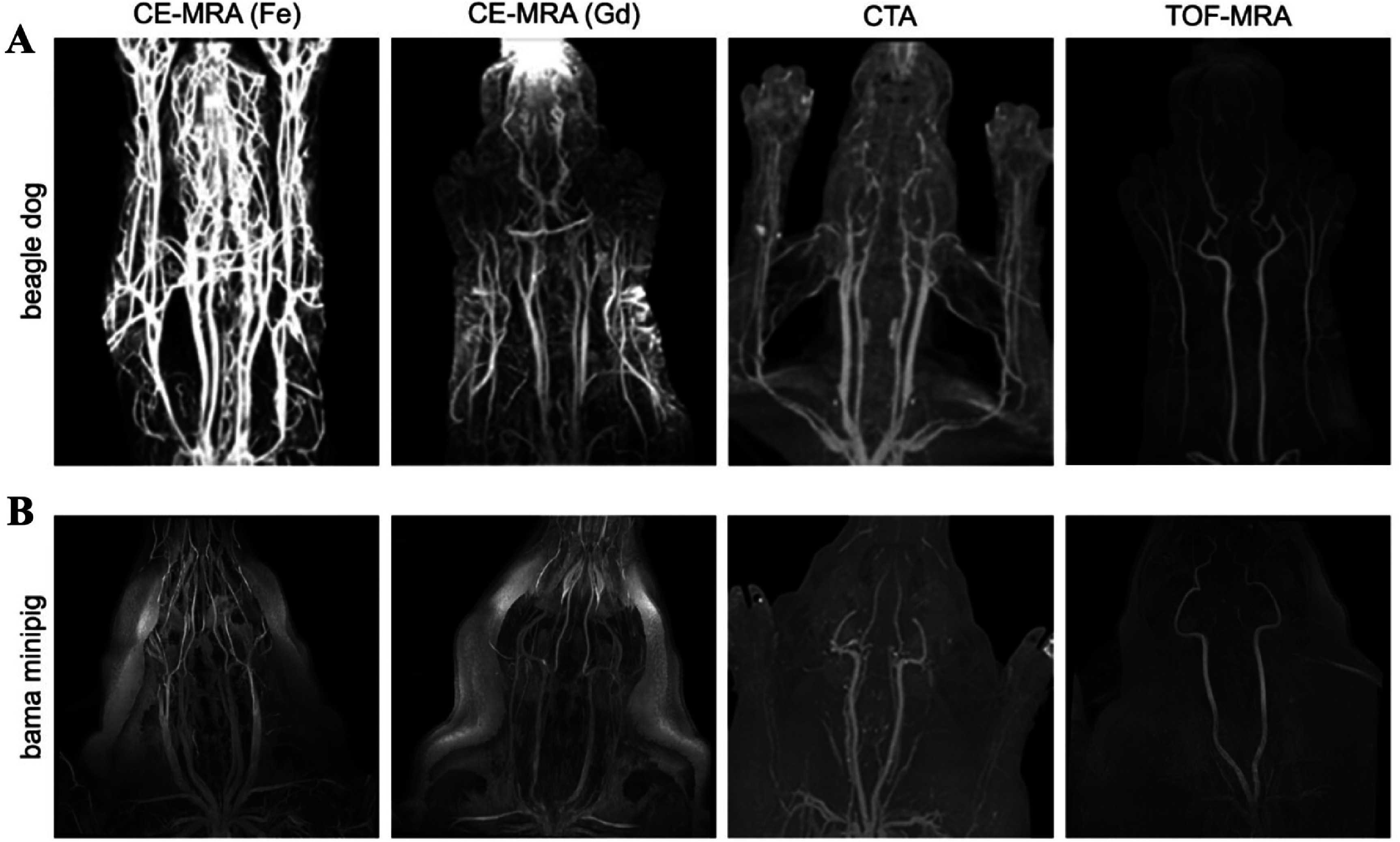
Maximum intensity projection (MIP) images of healthy beagle dogs (A) and Bama minipigs (B) acquired with four different imaging techniques. Reprinted with permission from [112]. Copyright (2025) American Chemical Society.

Afterward, recognizing that large-animal experiments are essential for evaluating new contrast materials and supporting clinical translation, they extended their study to beagle dogs and Bama minipigs (figures 11(A) and (B)). In these models, they performed head-and-neck angiography with PUSIONP-based Fe CE-MRA, conventional Gd CE-MRA, CTA, and TOF-MRA at multiple time points to compare vessel depiction and contrast kinetics. PUSIONP CE-MRA provided vascular boundary definition and lumen diameter measurements that were comparable or superior to CTA and TOF-MRA, while maintaining strong and persistent vascular enhancement over time. Taken together, these findings indicate that iron-oxide blood-pool agents such as PUSIONPs can now deliver clinical-grade vascular imaging performance, offering a realistic Gd-free alternative for MRA in both preclinical and translational contexts.

**Figure 12.**
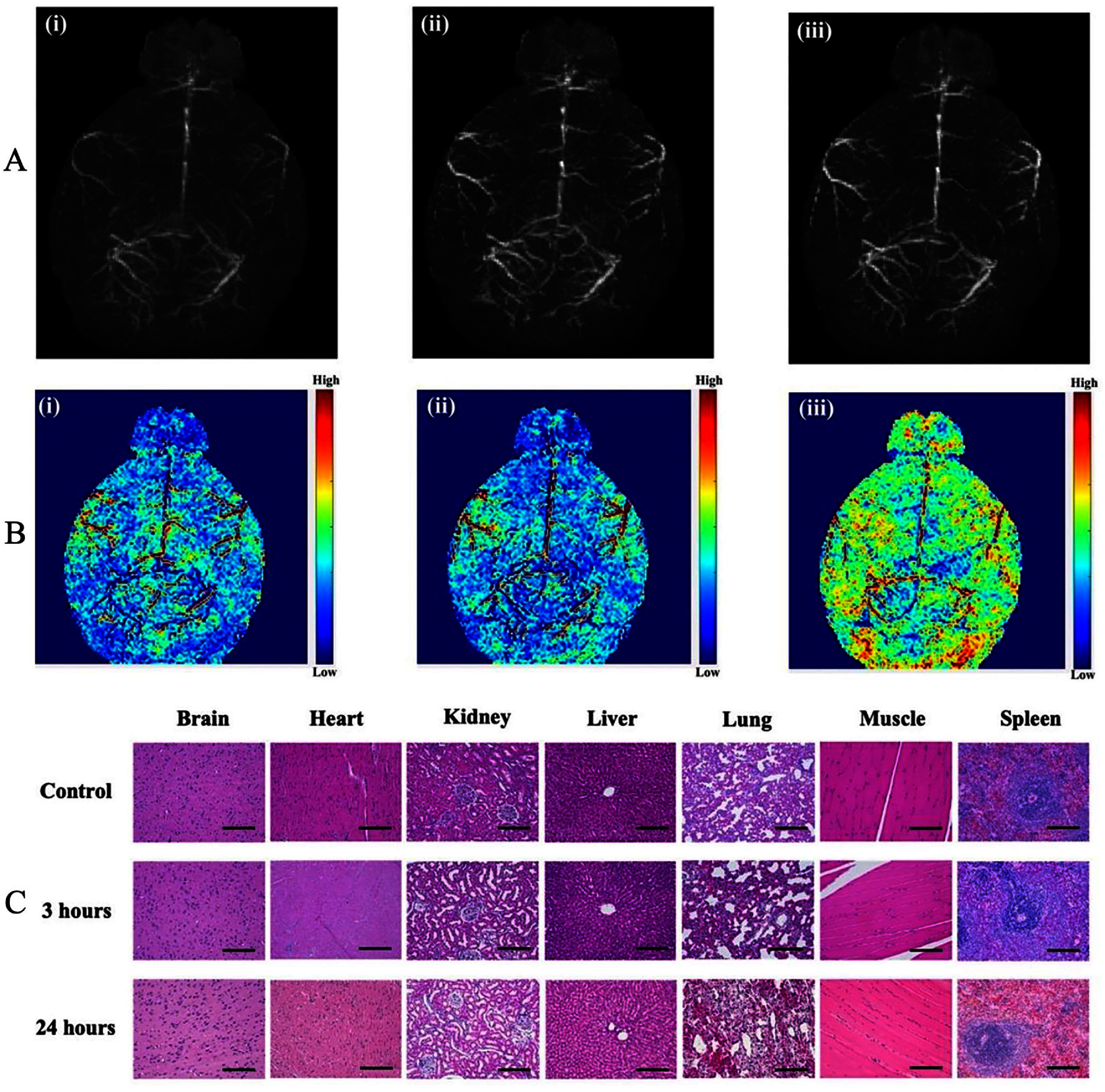
Cerebral angiography and safety evaluation using fluorescent magnetic protein nanoparticles (f-MPNPs). (A) Maximum-intensity-projection *T*_1_-weighted MR angiograms of the mouse brain acquired before f-MPNP injection (i) and at 5 min (ii) and 50 min (iii) post-injection, showing progressively brighter depiction of intracranial arteries and veins. (B) Corresponding color-coded maps of relative *T*₁-weighted signal intensity at the same time points, highlighting increased vessel-to-background contrast as f-MPNPs circulate. (C) Representative H&E-stained sections of major organs (brain, heart, kidney, liver, lung, muscle, and spleen) from control animals and from mice 3 h and 24 h after f-MPNP injection, showing no obvious acute histopathological abnormalities. Reproduced from [113] with permission from the Royal Society of Chemistry.

Mishra *et al* developed fluorescently tagged magnetic protein nanoparticles (f-MPNPs) as dual-modal contrast agents for ultra-high-field cerebral angiography [113]. Extremely small citric-acid-coated Fe_2_O_3_ cores (∼3.8 nm) were covalently linked to albumin and aggregated into cross-linked MPNPs (∼150 nm in diameter), which were subsequently labeled with Alexa 488, yielding a *T*_1_-dominant relaxivity profile (*r*_1_ ≈ 2.18 mM^−1^ s^−1^, *r*_2_/*r*_1_ ≈ 2.88 at 11.7 T) together with strong fluorescence. After intravenous injection in mice, f-MPNPs produced progressive brightening of intracranial arteries and veins on 3D-FLASH MRA, with vessel-to-tissue contrast clearly increasing between pre-contrast, 5 min, and 50 min scans (figure 12(A: i–iii)). Corresponding color-coded maps of relative signal intensity (figure 12(B: i–iii)) highlight both large arteries and smaller cortical vessels as the *T*_1_ enhancement develops. Wide-field fluorescence angiography resolved cortical capillaries below 40 *µ*m and matched the MR-visible vascular tree, confirming that the MRI and optical channels report the same cerebrovascular network. Biodistribution and H&E histology of major organs at 3 h and 24 h post-injection (figure 12(C)) showed efficient clearance via liver, spleen, and kidneys with no obvious acute tissue damage, indicating that protein-stabilized ultrasmall iron oxide formulations can provide Gd-free *T*₁ brain angiography with a complementary optical readout for neurovascular imaging.

### 3.2. Breast MRI

**Figure 13.**
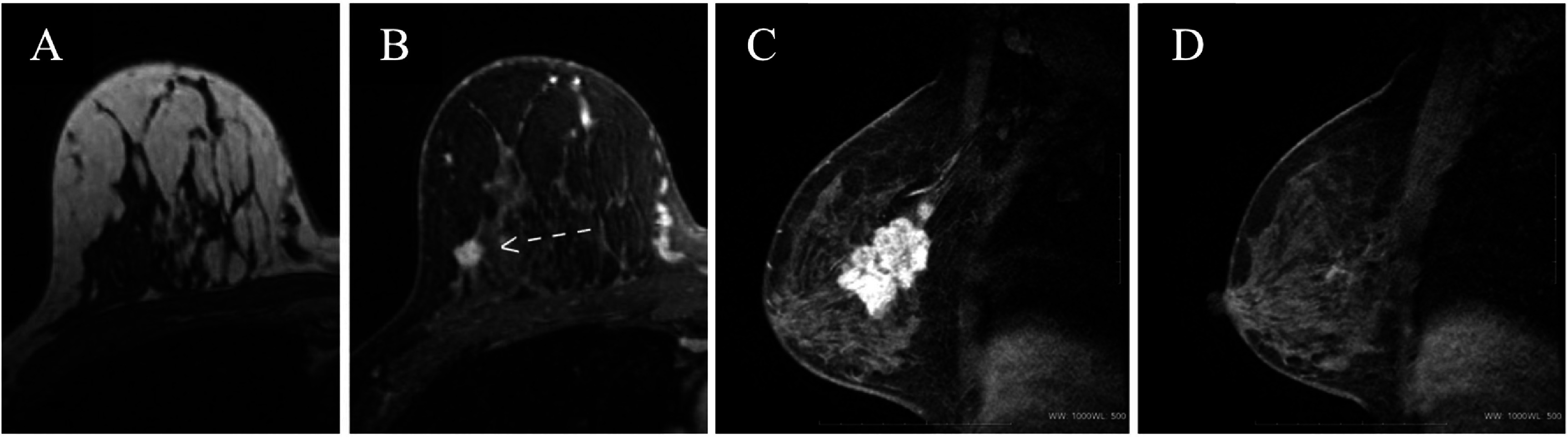
Breast cancer detection with MRI. (A) Cancer is difficult to see in the absence of a contrast agent. (B) Cancer is clear (arrow) when a contrast agent is used. A 53 yr-old female patient’s MRI images (with injection of Gd-DTPA) taken before (C) and after (D) NAC treatment demonstrate how the lesion decreased following treatment. (A) and (B) Reprinted from [123], Copyright (2005), with permission from Elsevier. (C) and (D) Reprinted from [124], Copyright (2015), with permission from Elsevier.

Breast cancer is the second most prevalent disease diagnosed in women and the second leading cause of cancer-related death in females [114]. Even though significant progress has been made in the therapy of breast cancer, creating imaging agents that can improve early diagnosis remains a difficult task. This is significant since improving the outcome of treatment and patient survival rates depends on the early identification of breast cancer [115]. It is important to note that tumor size is one of the most important factors in determining disease-free and cause-specific survival in invasive breast cancer, particularly in cases of node-negative breast cancers, where tumor size becomes crucial in determining the type and extent of subsequent surgical and oncological management [116]. In general, BCS, as opposed to mastectomy, provides the advantage of enhanced cosmetic satisfaction and quality of life for breast cancer patients, since the number of patients having mastectomies has increased steadily during the past 10 yr [117]. The high rate of mastectomies has led to the greatest proportion of patients needing organ reconstruction in general and some type of breast reconstruction in particular [118]. While x-ray mammography remains the dominant approach for breast cancer diagnosis, over the past 20 yr, methods of DCE and 3D lesion characterization in MRI have improved the sensitivity and specificity of breast cancer diagnosis [115]. MRI with contrast enhancement for the breast was created more than 25 yr ago [119]. DCE breast MRI, which is the most sensitive breast imaging tool, was made achievable with fast *T*_1_-weighted GE sequences. The initial findings were encouraging and revealed sensitivity and specificity values for the identification of breast cancer of above 95% [120]. Compared to ultrasound, mammography, or a combination of the two, this technology has been demonstrated to be more sensitive. In this regard, it is advantageous to have an MRI-guided breast biopsy because it enables the removal of non-palpable breast lesions that cannot be seen on other types of imaging, such as mammograms and ultrasound [121]. Also, it is crucial to have proper margin resection since the tumor-positive margin in BCS is strongly connected to the rate of breast cancer recurrence. Thus, MRI could be used to properly recognize the tumor’s location before surgery [117]. Regarding this, numerous initiatives have been made to enhance breast MRI’s diagnostic capabilities. Each of them aims to improve the distinction of contrast-enhancing lesions to further raise the specificity of breast MRI [122]. It has been demonstrated that DCE MRI of the breast is very sensitive in detecting invasive breast cancer and is not constrained by the density of the breast tissue (as can be seen in figures 13(A) and (B)). However, the breast’s natural MR contrast is insufficient on its own to distinguish between normal tissue and malignancy. To diagnose breast cancer with high accuracy, contrast agents must be administered intravenously.

In contrast to the vasculature of normal structures, cancerous tumors frequently have aberrant vessels that are leaky, allowing contrast to pool in the interstitial spaces and making the lesions evident on MRI. The primary strategy for separating benign from malignant lesions, as with other imaging modalities, is changes in lesion morphology. While benign lesions are often smooth, oval, or round, cancers typically have an uneven, speculated, or ductal appearance [123]. Thus, MRI is often used in conjunction with other breast imaging techniques, such as mammography and ultrasound, to provide a comprehensive assessment of breast health.

In Li *et al*’s study [124], a total of 21 pCR patients and 22 non-pCR randomly selected individuals who were undergoing NAC, and thereafter surgery were enrolled. Breast MRIs were performed on each patient both before and following chemotherapy. The two groups’ differences in diameter, area, and dynamic characteristics were compared between the initial and final MRIs. MRI tests were conducted using a specialized four-channel phased array breast coil in a 1.5 T MRI scanner. For dynamic acquisition, the vibrant sequence was repeatedly performed six times, using one pre-contrast and five post-contrast pictures. A saline flush was administered after the contrast agent (gadolinium-diethylenetriamine-pentaacetic acid, Gd-DTPA) was injected into the antecubital vein using a power injector at a rate of 2.0 ml s^−1^, depending on the patient’s body mass (0.2 mmol kg^−1^ ). Each patient had a breast MRI scan before and after the NAC. According to the data, MRI could distinguish between individuals with pCR and those without pCR by looking for changes in SIpeak (maximum signal intensity during vibrant sequences), diameter, and area before and after NAC (as can be seen in figures 13(C) and (D)).

**Figure 14.**
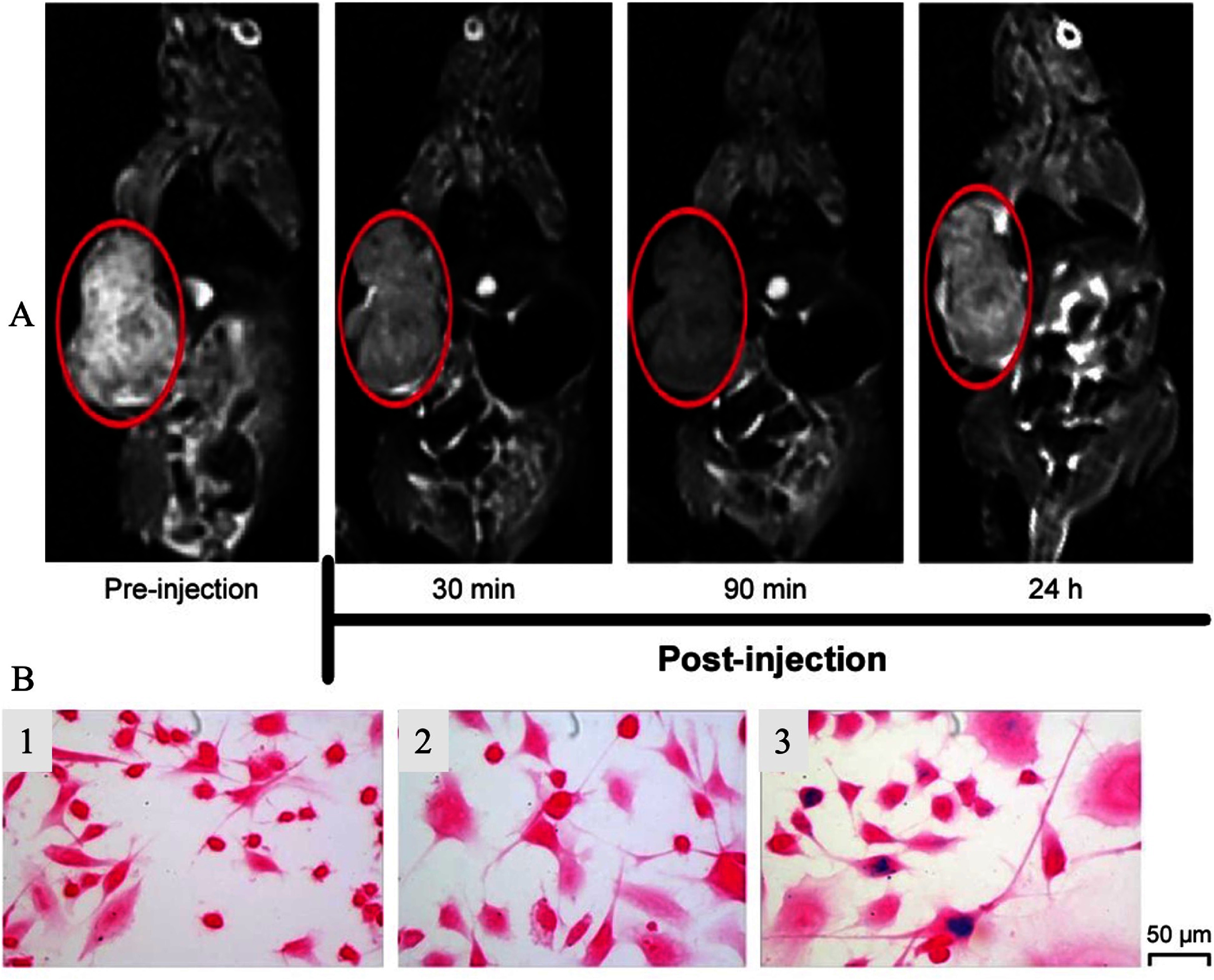
(A) *T*_2_-weighted MR images of MDA-MB-231 tumor (areas inside the red circle) bearing mice before and after intravenous injection with HA-SPIONs. (B) Prussian blue staining images of MDA-MB-231 cells; MDA-MB-231 cells only (1), MDA-MB-231 cells after 24 h of incubation with SPIONs (2), and MDA-MB-231 cells after 24 h of incubation with HA-SPIONs (3) [125]. (2017), reprinted by permission of the publisher (Taylor & Francis Ltd, www.tandfonline.com).

Yang *et al*’s research assessed the ability of HA-SPIONs (coated with hyaluronic acid, HA) to target breast cancer using MRI [125]. To examine the precise cellular uptake, HA-SPIONs were applied to CD44 HA receptors overexpressing MDA-MB-231 breast tumor cells. Furthermore, MRI of HA-SPIONs was studied in both *in vivo* and *in vitro* experiments. The intracellular presence of HA-SPIONs in MDA-MB-231 cells was shown via microscopy using Prussian blue staining (figure 14(B)). Using MDA-MB-231 tumor-bearing mice, the *in vivo* tumor-targeted MRI capabilities of HA-SPIONs as a *T*_2_-weighted MR contrast agent were studied, as shown in figure 14(A). Since HA-SPIONs shorten the transverse relaxation time *T*_2_ of surrounding water protons, they provide a considerable negative contrast enhancement in tumor tissues that darkens the tumor site on *T*_2_-weighted MR images. Therefore, in both *in vitro* and *in vivo* experiments, HA-SPIONs demonstrated a remarkable ability for MRI to recognize breast cancer cells.

### 3.3. Brain MRI

**Figure 15.**
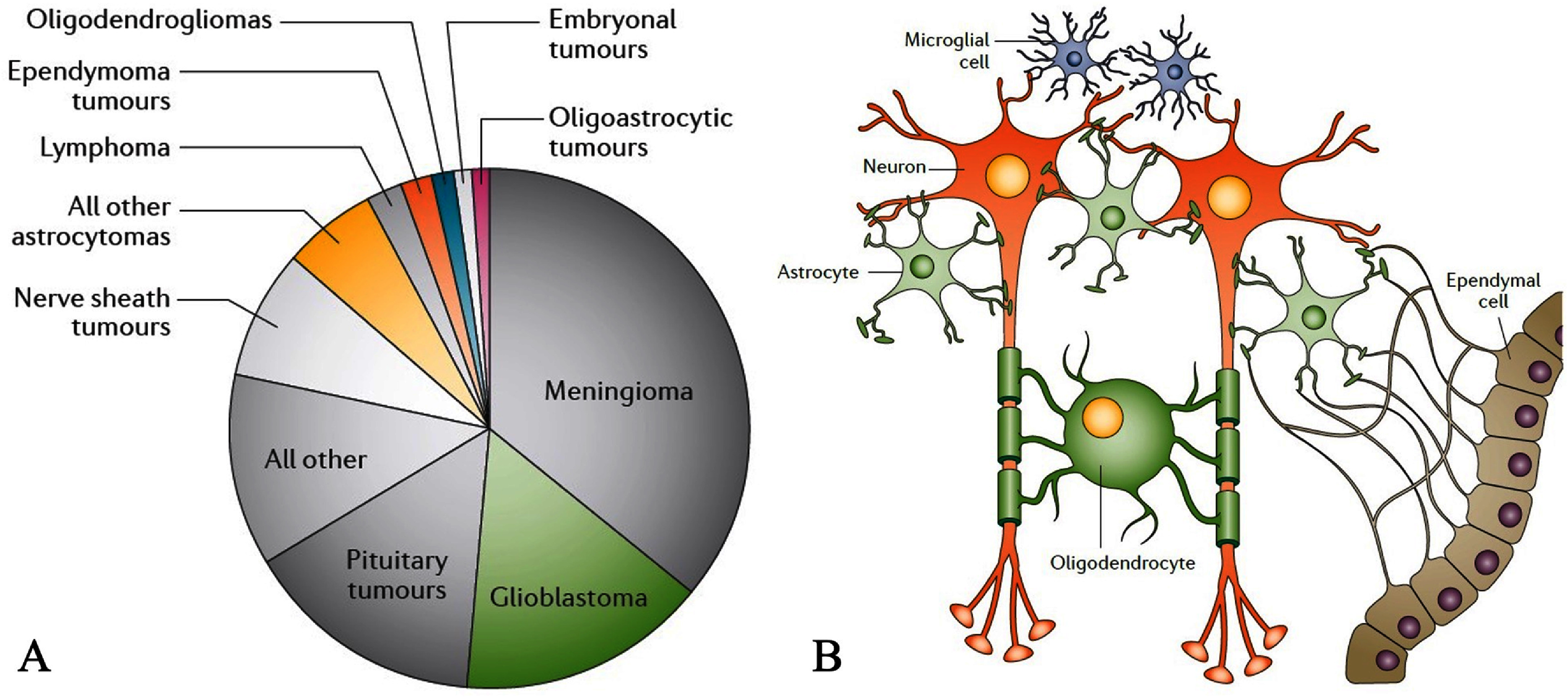
(A) Relative frequency of primary brain and central nervous system tumors, (B) Brain cells and brain tumors. Reproduced from [127], with permission from Springer Nature.

Understanding how the human brain develops is one of the most intriguing scientific challenges. Early in embryonic life, brain cells grow and mature in a precise sequence, leading to approximately 100 billion neurons at birth. A newborn’s brain is only a fifth of its adult size, but it grows rapidly through genetic programming and environmental influences. MRI technology allows us to observe these developmental processes in detail, providing valuable insights into how the brain matures over time [126]. On the other hand, brain tumors are associated with a high rate of morbidity and mortality because they are often localized and exhibit locally invasive growth. The majority of neoplastic brain lesions are metastases that result from extranervous system cancers, which are five to ten times more prevalent than original brain tumors [127]. The two most prevalent forms of primary brain tumors are gliomas and meningiomas, as statistically summarized in figure 15(A). Nearly 30% of primary brain tumors and 80% of malignant ones are gliomas. Based on their morphological resemblance to the neuroglial cell types present in the normal brain, they are histologically classified as astrocytomas, oligodendrogliomas, mixed oligoastrocytic gliomas, or ependymomas (see figure 15(B)).

One of the most popular imaging techniques used to evaluate brain malignancies is MRI. The anatomical location and morphological features of brain tumors and metastases have been extensively determined using qualitative *T*_1_ and *T*_2_-weighted imaging, especially post-Gd *T*_1_-weighted images. The different *T*_1_ and *T*_2_ signal intensities can be statistically mapped to detect pathophysiological changes since they are based on variations in the intrinsic relaxation times of the brain tissue related to acquisition settings [128].

**Figure 16.**
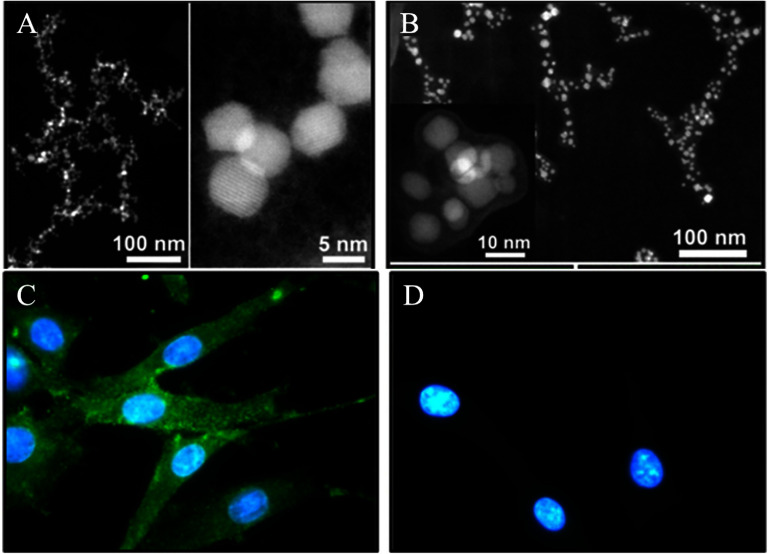
Images of BSA-coated (A) and cross-linked BSA-coated MNPs (B). Immunofluorescent analysis of MNP-BSACl-mAbVEGF (C) and MNP-BSACl-IgG (D) on the fixed culture of glioma C6 cells. Reprinted from [129], Copyright (2015), with permission from Elsevier.

The study by Abakumov *et al* [129] focuses on the synthesis and characterization of targeted MNPs designed for *in vivo* glioma visualization using MRI. Iron oxide (Fe_3_O_4_) cores were synthesized via thermal decomposition and subsequently coated with BSA, resulting in nanoparticles with an effective diameter (*D*_eff_) of 53 ± 9 nm. To enhance colloidal stability, the BSA coating was cross-linked, and monoclonal antibodies against vascular endothelial growth factor (mAbVEGF) were covalently attached via a PEG linker (figures 16(A) and (B)). They then assessed the *in vitro* targeting ability of the nanoparticles to their receptor using C6 glioma cells, which express membrane-bound VEGF. Immunofluorescence analysis indicated that MNP-BSACl-mAbVEGF nanoparticles successfully bound to C6 glioma cells (figure 16(C)). In contrast, MNP-BSACl-IgG nanoparticles showed no binding (figure 16(D)).

**Figure 17.**
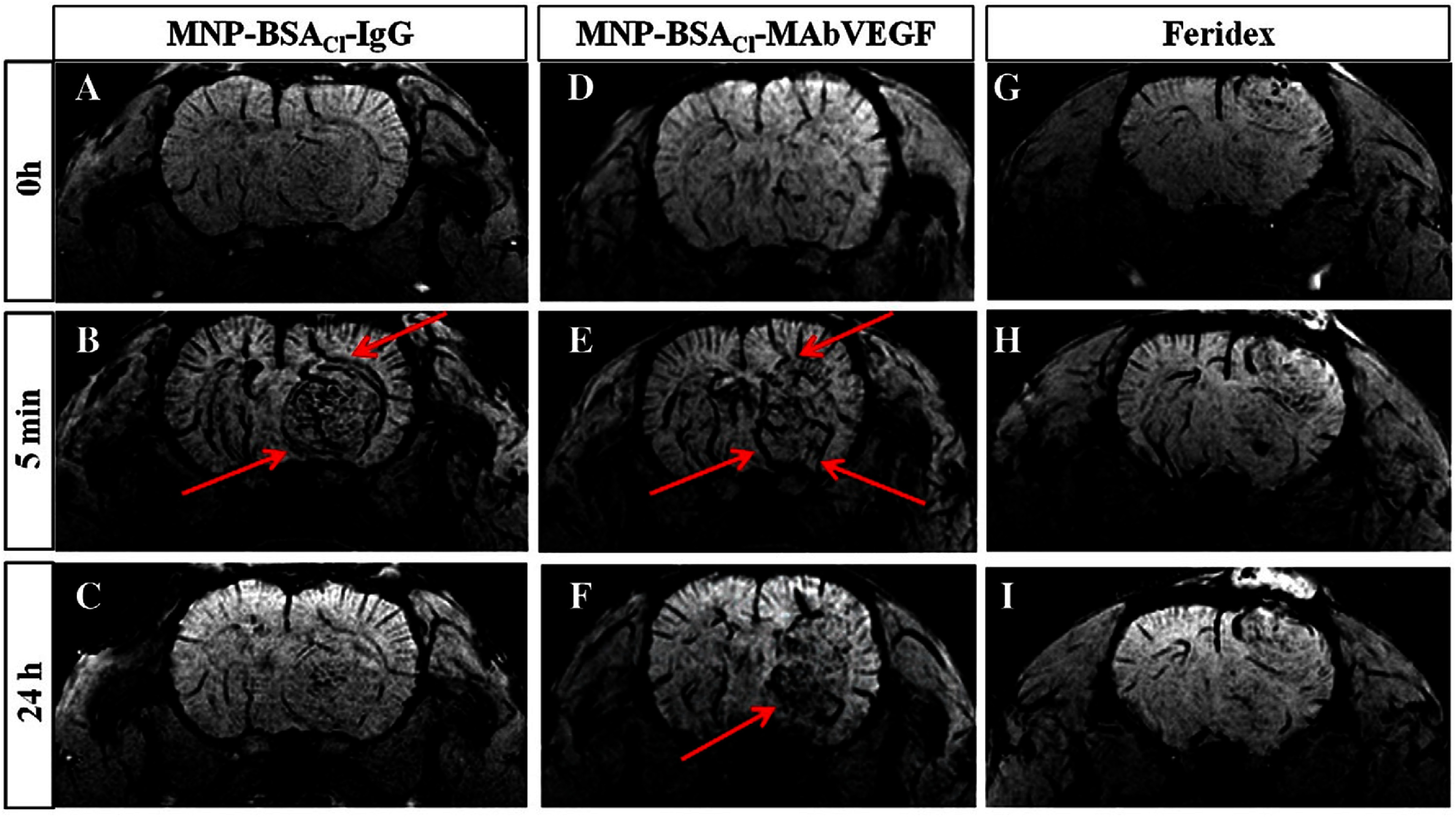
SWI MR images of rats with experimental C6 glioma taken before (A), (D), (G) and 5 min (B), (E), (H) and 24 h (C), (F), (I) after intravenous (i.v.) injection of MNP-BSA_Cl_-IgG (left column), MNP-BSA_Cl_-mAbVEGF (middle column), and Feridex (right column). Red arrows indicate the glioma (tumor) region, particularly tumor-associated vessels highlighted after nanoparticle administration. Reprinted from [129], Copyright (2015), with permission from Elsevier.

After that, using intravenous administration of MNP-BSA_Cl_-mAbVEGF, MNP-BSA_Cl_-IgG, and Feridex at equal iron oxide dosages, they performed MRI visualization of cerebral gliomas in rats. After injection, MNP-BSA_Cl_-IgG allowed for the immediate visualization of tumors and tumor vessels (figure 17(B)). However, its plasma concentration rapidly decreased, resulting in deteriorated contrast after 2 h and a negligible signal at 24 h, making it impossible to distinguish between treated and non-treated animals (figures 17(A) vs (C)). Similarly, the early MRI images following MNP-BSA_Cl_-mAbVEGF injection (figure 17(D)) showed limited initial contrast enhancement. The tumor signal at 5 min and 2 h for VEGF-targeted MNP-BSA_Cl_-mAbVEGF was comparable to that of MNP-BSA_Cl_-IgG (figure 17(E)). MRI contrast, however, was still much higher than the control at 24 h after injection (figure 17(F)), suggesting tumor accumulation. On the other hand, Feridex was unable to detect gliomas in these circumstances, most likely because of quick removal (figures 17(G)–(I)).

## 4. Biocompatibility and safety concerns for contrast agents

The potential for nanomaterials to induce toxicity when interacting with biological systems is a significant concern when considering their use for *in vivo* applications [130]. CE-MRI has been widely used for clinical and preclinical research, and it is estimated that Gd, Mn, and iron oxide nanoparticles have formed the basis for clinically employed contrast agents [131]. GBCAs have advanced the diagnosis and treatment of disease by providing much-needed image augmentation in MRI. These developments have been enabled by ionized Gd’s paramagnetic properties, although there remains a risk of cytotoxicity. Organic chelates were used in the formulation of GBCAs to lessen the potential for toxicity from unbound Gd ions. Since Gd has substantial paramagnetic retention, it is recommended for MRI enhancement, even though it is not a typical constituent of human tissue like the trace element Mn [132]. However, because Gd and Mn chelates are rapidly excreted from the body, they typically have short blood circulation times. As a result, high injection doses are required to achieve the desired detection levels, which may increase the risk of biotoxicity due to the release of free metal ions into the body.

For example, in patients with severe renal impairment, free Gd^3+^ ions can induce nephrogenic NSF [133–135], a rare disorder that often arises after renal failure or liver transplantation [136], causing skin thickening, darkening, and impaired heart and lung function. Furthermore, recent studies have shown that Gd chelates may accumulate in the brain after repeated use, raising concerns about potential neurotoxicity [137]. Additionally, while manganese is essential for human health in trace amounts and has no established link to NSF, excessive exposure to free Mn ions can lead to Manganism, a neurodegenerative disorder resembling Parkinson’s disease symptoms such as tremors, muscle rigidity, and cognitive impairment [78, 131].

Through extensive research, researchers have recognized SPIONs as valuable complements to conventional MRI contrast agents, enhancing imaging capabilities for specific applications. SPIONs offer excellent relaxometric properties, active targeting abilities, and potential for multimodal imaging and theranostics. Importantly, nanoscale iron oxides are widely regarded as safer alternatives to traditional agents, such as Gd-based nanomaterials, due to their demonstrated biocompatibility in numerous *in vitro* and *in vivo* studies. After internalization by macrophages, SPIONs degrade into iron ions that integrate into the body’s natural iron metabolism, further supporting their safety and biodegradability [138]. Additionally, iron oxide nanoparticles, ranging in size from 1 to 1000 nm, offer unique advantages for crossing the BBB. Studies indicate that smaller nanoparticles exhibit greater permeability through BBB gaps, while particles larger than 200 nm demonstrate minimal penetration. However, nanoparticles smaller than 5 nm are rapidly cleared from circulation via renal filtration, reducing their blood retention time. As a result, most studies focus on nanoparticles between 10 and 100 nm, which optimize BBB permeability while maintaining systemic circulation [139, 140].

Despite these advantages, achieving clinical success with SPIONs requires addressing several challenges. Key hurdles include ensuring long-term colloidal stability, optimizing biological interactions, extending circulation times, achieving large-scale reproducible synthesis, and managing production costs [141, 142]. Another major challenge for SPIONs lies in developing surface coating materials that not only stabilize nanoparticles but also provide active functional groups for controlled bioconjugation of probe ligands. The surface chemistry strongly affects the relaxivity of SPIONs by either excluding water molecules from the magnetic core or increasing their residence time through hydrogen bonding with ligands; therefore, careful optimization of surface ligands is essential to achieve the desired imaging performance [141].

Superior SPIONs with consistent size and good crystallinity are often synthesized in hydrophobic organic solvents, but their poor stability in aqueous conditions limits biological applications. To address this, surface modification strategies are employed to impart hydrophilicity, improve colloidal stability, and minimize toxicity. Two common approaches include ligand exchange with hydrophilic molecules and encapsulation within biocompatible shells. Suitable ligands facilitate phase transfer from organic to aqueous media by binding to the nanoparticle surface, while shells made from materials like polymers or silica offer robust stabilization [64, 143]. Numerous synthetic and natural polymers have been widely applied in biomedical fields. Clinically used polymers for immobilizing MNPs, such as PLGA, PLA, PGA, PEG, dextran, and chitosan, contribute to minimizing toxicological concerns and improving safety [93]. These advancements in nanoparticle surface engineering have been pivotal in facilitating the clinical translation of SPIONs. Following the development of more stable and biocompatible formulations, several SPION-based contrast agents progressed to regulatory approval and clinical application.

Recognizing the potential of SPIONs, the FDA approved the first commercially available iron oxide nanoparticle solution in 1996. Standard SPIONs, with a hydrodynamic size of 40–150 nm and coated with dextran, were developed for liver imaging and included formulations such as ferumoxide (Endorem®/Ferridex®) and ferucarbotran (Resovist®). One unexpected side effect associated with Ferridex® was severe back pain following bolus injection. Due to limited demand and several adverse effects, Ferridex® was withdrawn from the market in 2011, while Resovist® remains available only in a few countries, including Japan. In 2011, additional SPIO-based agents were introduced, such as ferumoxtran (Combidex®), initially used for imaging lymph node metastases in prostate cancer, and ferumoxytol (Feraheme®), developed to treat iron deficiency anemia in patients with chronic kidney disease. However, ferumoxytol has been associated with certain adverse effects, leading the FDA to issue a black box warning due to the risk of potentially life-threatening anaphylactic or hypersensitivity reactions. Despite these risks, ferumoxytol remains one of the few SPION formulations still in clinical use, both for its original purpose and off label as an MRI angiography agent for patients who cannot tolerate gadolinium. Notably, several formulations, including ferristene (Abdoscan®), ferumoxsil (GastroMARK® in the EU and Lumirem® in the USA), have been utilized as oral gastrointestinal contrast agents. These are classified as safe and effective, are typical SPION formulations, and feature coatings of insoluble compounds (polystyrene for ferristene and siloxane for ferumoxsil). Currently, ferumoxsil is the only iron oxide nanoparticle approved by the FDA for imaging purposes [15, 137, 144].

Taken together, the clinical trajectory of Fe_3_O_4_-based contrast agents illustrates both the promise and the limitations of magnetic iron oxide-based nanoparticles in MRI. Several dextran- or polymer-coated SPIONs formulations have achieved regulatory approval and demonstrated good diagnostic performance in liver, lymph node, vascular, and gastrointestinal imaging, and most withdrawals have been driven by commercial or strategic considerations rather than unequivocal toxicity signals [15, 137, 144–146]. At the same time, the fact that only a few iron oxide agents remain in routine clinical use, despite an extensive preclinical literature on SPIONs, highlights persistent translational barriers, including manufacturing complexity, cost, and hypersensitivity reactions in susceptible patients, and competition from gadolinium-based agents. In practice, Gd-based chelates remain the dominant clinical MRI contrast agents not only because of their strong and predictable *T*_1_ shortening, but also because radiologists are more comfortable interpreting bright *T*_1_ enhancement than the negative *T*_2_ signal loss and susceptibility artifacts produced by classical IONPs, especially in organs with intrinsically low signal or haemorrhage. Moreover, most approved IONP formulations have suffered from low utilization and limited financial return, so that several agents were withdrawn for economic and strategic reasons despite acceptable safety profiles [132, 147–149]. As a result, any new IOPNs formulation must offer not only acceptable safety and pharmacokinetics but also a clear diagnostic or theranostic advantage if it is to displace GBCAs in standard workflows. So, future development of clinically viable IOPNs contrast agents will therefore depend not only on favorable relaxivity and biocompatibility, but also on robust large-scale synthesis, predictable pharmacokinetics, and clear added value over existing Gd-based products.

In summary, after more than 30 yr of clinical application, CE-MRI has proven to be an invaluable diagnostic imaging modality, surpassing initial expectations and establishing itself as a crucial tool for disease diagnosis and management worldwide. CE-MRI continues to evolve, with advancements in techniques, innovative equipment, and new contrast agents paving the way for more targeted and sensitive imaging, improved patient care, and addressing related clinical challenges [119].

## 5. Conclusions and perspectives

MRI has become a vital tool in biomedical research and clinical diagnosis, and its technical evolution now directly shapes the requirements placed on contrast agents. Over recent decades, MRI has progressed from purely anatomical imaging to a multimodal platform that incorporates functional, perfusion, diffusion, and quantitative mapping techniques, enabling non-invasive probing of tissue physiology and microstructure. As MRI methods become more advanced, contrast agents are expected not only to make lesions easier to see but also to provide reliable quantitative measurements that can be compared across different organs, scanners, and field strengths. In this review, we first outline the MRI principles and relaxation mechanisms most relevant for contrast-agent design and then discuss how MNP systems perform in key clinical application areas such as cardiac, breast, and brain MRI. We summarized how modern clinical MRI is applied to organs such as the brain, breast, and cardiovascular system, and used these organ-specific examples to illustrate how MNP properties (core size, composition, and surface coating) influence *in vivo* relaxivity behavior and imaging performance in vascular and tissue targets. Taken together with the biocompatibility and regulatory considerations reviewed in section 4, this provides a coherent overview linking key MNP design parameters to both contrast behavior and major translational constraints. Since the first clinical introduction of MRI contrast agents in the 1980s, systems-level improvements in data acquisition, image reconstruction, and hardware design have continuously enhanced spatial and temporal resolution. Innovative technologies from fields such as semiconductors, computer science, and data processing have been rapidly integrated into MRI, allowing it to generate large functional and anatomical datasets that serve as crucial tools across diverse research domains [150]. More recently, quantitative MRI, high-field systems, and advanced reconstruction methods have further increased sensitivity to subtle changes in tissue properties, thereby tightening the constraints on contrast-agent pharmacokinetics, relaxivity, and safety profiles. In parallel, contrast-agent development has evolved from the first generation of small-molecule Gd chelates, such as Gadopentetate dimeglumine (Magnevist®), toward macrocyclic, higher-relaxivity, and lower-retention formulations in response to safety concerns, including NSF and brain deposition [151]. As a result, there remains an urgent need for contrast agents that are more selective, offer stronger signal enhancement, and exhibit improved biocompatibility and clearance profiles. At the same time, as discussed in section 4, the clinical trajectory of SPION-based agents shows that favorable biocompatibility and liver–spleen imaging performance are not, by themselves, sufficient for durable adoption, given competition from well-established Gd chelates and the relatively limited clinical demand for their specialized indications. Considering these clinical and regulatory realities, the question is no longer whether SPIONs will globally replace gadolinium chelates, but rather under what conditions they genuinely add value. Safety concerns around GBCAs, together with the mixed market history of SPION-based agents, have shifted the focus from simple signal enhancement toward indication-specific design: which clinical problems justify more complex nano-architectures, and which situations can still be adequately addressed with conventional small molecules. This more pragmatic view frames current research on organ-targeted and theranostic formulations, particularly in applications where repeated dosing, need for molecular specificity, or integration with therapy make nanoparticle-based approaches especially attractive [119]. Within this landscape, SPIONs stand out not as universal replacements for Gd chelates, but as platforms that can be rationally engineered for specific niches: molecular or cellular targeting, tunable pharmacokinetics via surface chemistry, and built-in therapeutic functions such as hyperthermia [85] or drug delivery. In this sense, the most realistic future for SPIONs lies in well-defined, high-value indications where their modular design can deliver capabilities (e.g. targeted, quantitative, or theranostic readouts) that conventional small-molecule agents cannot easily provide.

Taken together with the safety and regulatory considerations outlined above, these trends imply that future contrast-agent design must simultaneously balance three elements: (i) safety and long-term tolerability, including minimized metal retention and hypersensitivity risk; (ii) robust, preferably quantitative imaging performance compatible with high-field and advanced pulse sequences; and (iii) the ability to interface with complex biological pathways or therapeutic functions when needed. From the contrast-agent perspective, current trajectories do not point to a simple replacement of Gd-based chelates by SPIONs, but rather to a diversified ecosystem in which macrocyclic Gd agents remain the backbone of routine clinical MRI, SPIONs are developed for indications where they offer clear incremental value (e.g. blood-pool imaging, cell tracking, or image-guided interventions). In this context, iron-based MNPs evolve from *T*_2_-darkening SPIONs into more specialized platforms: ultrasmall and surface-engineered iron oxides for *T*_1_ or *T*_1_/*T*_2_ hybrid contrast, targeted formulations for inflammatory and oncologic imaging, and agents specifically optimized for high-field, quantitative MRI protocols. Moreover, regulatory and safety frameworks will increasingly require systematic data on long-term biodistribution, metal release, and immunological responses, together with standardized relaxivity measurements under clinically relevant conditions, before new nanoparticle agents can progress beyond early-phase trials. Within this framework, the most distinctive opportunity for MNPs lies in their ability to act not only as imaging probes but also as functional components of therapeutic systems. Beyond pure diagnostics, MNPs are also poised to play a central role in theranostic and multimodal strategies over the next 10 yr. The same iron-oxide cores used for MRI can serve as heat mediators for magnetic hyperthermia, enabling single-injection formulations to support tumor detection, drug delivery, image-guided therapy, and treatment monitoring within a unified platform. Conceptually, such systems could act as single-dose nanoparticle theranostics, in which the same MNPs are first used to localize disease with MRI, then activated for hyperthermia or controlled drug release, and finally followed longitudinally to assess response. Integration of MRI, radiomics, and emerging AI-based analysis has the potential to improve diagnostic sensitivity and specificity, particularly for heterogeneous tumors in oncologic imaging. Real progress, however, will require closer coordination between materials science, imaging physics, and clinical trial design so that nanoparticle contrast agents are developed with clearly defined clinical questions and realistic paths to approval in mind. If these scientific, regulatory, and practical challenges can be addressed, MNPs are well positioned to evolve from predominantly experimental tools into a small but strategically important class of clinically relevant MRI contrast agents with roles both in diagnosis and in image-guided therapy.

## Data Availability

No new data were created or analysed in this study.
